# Hedonic quality or reward? A study of basic pleasure in homeostasis and decision making of a motivated autonomous robot

**DOI:** 10.1177/1059712316666331

**Published:** 2016-10-12

**Authors:** Matthew Lewis, Lola Cañamero

**Affiliations:** Embodied Emotion, Cognition and (Inter-)Action Lab, School of Computer Science, University of Hertfordshire, UK

**Keywords:** Action selection, embodied autonomous robots, homeostasis, hormonal modulation, motivation and emotion, pleasure

## Abstract

We present a robot architecture and experiments to investigate some of the roles that pleasure plays in the decision making (action selection) process of an autonomous robot that must survive in its environment. We have conducted three sets of experiments to assess the effect of different types of pleasure—related versus unrelated to the satisfaction of physiological needs—under different environmental circumstances. Our results indicate that pleasure, including pleasure unrelated to need satisfaction, has value for homeostatic management in terms of improved viability and increased flexibility in adaptive behavior.

## 1 Introduction

We present a biologically-inspired robot architecture and experiments to investigate some of the roles that pleasure plays in the decision making or action selection (AS) process of a motivationally autonomous robot that must survive or remain ‘viable’ (Ashby, 1960) in its environment. This study is framed by our long-term interest in the interactions between emotion, motivation and cognition from an embodied perspective, and is a first step towards a more systematic study of the roles of pleasure in such interactions.

Many definitions of pleasure have been provided in the literature, reflecting the fact of its multi-faceted nature, its multiple meanings, and its multiple underlying biological mechanisms, to the extent that some authors talk about ‘pleasures’ rather than ‘pleasure’ as a single notion (Frijda, 2010; Kringelbach & Berridge, 2010), e.g. sensory pleasure, non-sensory linkings, pleasures of achievement, pleasures of gain and relief, social pleasure, activity pleasures, esthetic pleasures. Underlying the different views, however, we can identify a common element of ‘positive affect’, of enjoyment, which is also found in lay, non-technical definitions (e.g. in dictionaries such as the Merriam–Webster or the Oxford). In this paper, we take such a broad, common-sense definition of pleasure as ‘liking’, focusing on two different contexts in which such ‘liking’ can take place: as linked to the satisfaction of survival-related physiological needs, and as a purely hedonic quality not directly linked to need satisfaction. In both cases, we have focused our study on the influence that pleasure has on the perception of external stimuli, and the underlying mechanism we have adopted to model pleasure is a simulation of hormonal modulation of perception. Unlike related work in robotics (Krichmar, 2008, 2012, 2013; Sporns & Alexander, 2002), our simulated hormone constitutes an abstract model aimed to capture the (gross) dynamics of modulation rather than modeling the behavior of specific chemicals underlying pleasure and more generally affective phenomena. Hormonal modulation has been used in robots for other purposes, such as behavior control (Moioli, Vargas, & Husbands, 2009), learning based on value and reward systems (e.g. the 2013 special issue of *Frontiers in Neurorobotics* on this topic; see (Krichmar and Röhrbein, 2013) for a review of the papers), to model intrinsic motivation (Kaplan & Oudeyer, 2007; Luciw, Kompella, Kazerounian, & Schmidhuber, 2013), energetic autonomy (Lowe et al., 2010; Sauzé & Neal, 2010), and evolutionary and modular robotics (Hamann, Stradner, Schmickl, & Crailsheim, 2010).

Although pleasure is intimately related to affect, and more specifically to emotion, the nature of this relationship remains elusive. Some consider pleasure as an emotion—e.g. one of the earliest forms of emotion that evolved (Panksepp, 1998)—others think that pleasure, even though at the origin of emotions, is not an emotion itself but ‘a constituent quality of certain emotions as well as a trigger for certain emotions’ (Damasio, 1999, p. 76). Firmly grounded in the body’s biological ‘machinery’ at different levels, the term ‘pleasure’ is normally used to denote a *subjective quality*: a positive hedonic quality, an affective sensation or feeling of pleasantness. To some, pleasure has both sensory and affective elements (Nafe, 1924), whereas for most, pleasure is *the* affective component (Cabanac, 2010; Kringelbach, 2010), a ‘gloss’ (Frijda, 2010) of sensory processing.

Pleasure is often associated with positive valence. However, the interactions between hedonic feelings, positive affect, and approach behavior can be very complex (Leknes & Tracy, 2010), and their underlying mechanisms are not clear. This link is often explained in terms of the biological ‘utility’ (e.g. adaptive value, evolutionary usefulness) of pleasure, which, from this perspective, would signal that stimuli are beneficial (Cabanac, 1971, 1979; Panksepp, 1998) and foster the acceptance of such stimuli (Frijda, 1986). This view is particularly compatible with models of the body in terms of homeostasis (Leknes & Tracy, 2010), as well as with models of learning inspired by classic and operant conditioning (Rolls, 2014), and is reflected by their focus on the ‘reward’ aspect of pleasure to the neglect of other aspects. This is also the predominant interest of robotic models that take into account pleasure, mostly in the context of reinforcement learning (e.g. Cos, Cañamero, Hayes, & Gillies, 2013; Gadanho & Hallam, 2001; Hiolle & Cañamero, 2009; Kitano, 1995). In previous work, we have investigated the role of pleasure as reward in the maintenance of homeostasis in a reinforcement learning model (Cos et al., 2013). In this paper, we depart from the idea that pleasure is necessarily linked with reward—in the same way as value is not necessarily linked with reward (Krichmar & Röhrbein, 2013)—or with signaling biological usefulness, opening the door to the investigation of the role of other types of pleasure not directly related with the satisfaction of needs (Frijda, 2010), in addition to pleasure stemming from need satisfaction.

Thinking about the link between pleasure and valence leads us to a key unresolved question in affective neuroscience and psychology: how do we go from ‘liking’ something to ‘wanting’ it? This link is, once more, often conceptualized in terms of ‘usefulness’ and ‘reward’ and, in the latter case, in the context of learning. Again, in this paper we depart from this view, in two respects. First, we think that hedonic quality (just ‘liking’, ‘pure pleasure’ unrelated to need satisfaction) might also have an important role in preference behavior and motivation (Frijda, 1986, 2010; Young, 1961). Second, given the involvement of pleasure in basic life regulation in line with its early evolutionary origins (Damasio, 1999; Panksepp, 1998), we think that, to better understand pleasure, we need to consider its roles in the context of ‘simpler’ cognitive functions, and notably in the context of perception, since pleasure involves closely related hedonic and sensory aspects. We have thus investigated the effect of pleasure on the perception of external stimuli and, more specifically, of the stimuli relevant to the satisfaction of homeostatically-controlled internal needs. As we will see, in our model, the modulation of motivation-related perception through pleasure provides a link between ‘liking’ and ‘wanting’, as it changes the ‘attentional effort’ (Sarter, Gehring, & Kozak, 2006) or ‘incentive salience’ (Berridge & Robinson, 1998) of the stimuli. As Pessoa (2013, p. 190) puts it: ‘At the perceptual level, items with affective/motivational content act as if they had increased *salience*, which improves performance if they are task relevant but impairs it if they are task irrelevant.’

In previous work (Cañamero & Avila-García, 2007), we had used hormonal modulation of the perceptual element of motivation as a function of increased internal deficits and the presence of threats in the environment, i.e. hormone was released signaling that *things were not functioning well*. In this paper, a simulated modulatory pleasure hormone is released as a function of need satisfaction, signaling an improvement in the interaction with the environment and fosters ‘openness’—increasing the interaction with the environment or continuing an interaction that is going well. We thus follow the principle that pleasure *signals well functioning* (Panksepp, 1998).

To facilitate systematic analysis of experimental results, we have tested our model of pleasure using what in the AS literature is known as a ‘two-resource problem’ (2RP) (Spier & McFarland, 1997), implementing the simplest decision-making scenario. As its name suggests, in this scenario, an agent (animal or robot) must autonomously decide which of the two resources available in the environment it should consume in a timely fashion in order to satisfy its two survival-related needs successfully. The experiments reported here aim to compare the viability and behavior of robots whose pleasure hormones, released under different circumstances, play different roles. We compare the effects of pleasure that varies as a function of need satisfaction with the effects of pleasure as pure hedonic quality, unrelated to need satisfaction—either independent or added to it. In all cases, the pleasure hormone acts on the ‘subjective assessment’ or ‘assignment of value’ to the perceived stimuli, modifying their incentive salience.

## 2 Robot’s action selection architecture

**Figure 1.**
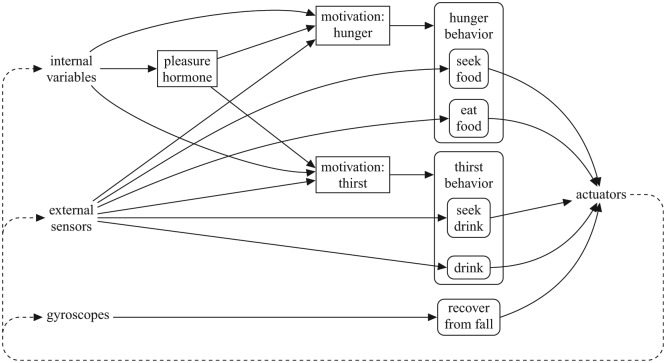
The AS architecture of our robot. Rounded boxes represent behaviors, square boxes represent other internal elements. Composite behaviors are shown in compact form. Some behaviors (‘return-to-neutral’) are omitted to avoid cluttering the diagram.

Our robot’s architecture (Figure 1) follows design principles of embodied artificial intelligence (Brooks, 1991; Pfeifer, Iida, & Bongard, 2005; Steels, 1993), and builds on our longstanding approach (Cañamero, 1997, 2001) to ground embodied cognition and interaction in ‘core’ affect modeled around a ‘physiology’ of homeostatically-controlled essential variables. For this study, we have used the humanoid robot Nao, since we have developed our pleasure model with the intention to implement it in the autonomous social robot toddler Robin based on a Nao robot, (www.emotion-modeling. info/robin) (Cañamero & Lewis, 2016; Lewis & Cañamero, 2014) that we started developing as part of the EU project ALIZ-E.

As a basis for our architecture, we developed a framework in UrbiScript (Baillie, Demaille, Hocquet, Nottale & Tardieu, 2008) consisting of base classes for behaviors, and functions to execute (either synchronously or asynchronously, as required) one or more behaviors of different types, e.g. with different levels of granularity and structure, different constraints, and different durations and temporal dynamics.^1^

### 2.1 Physiology

To implement a simple 2RP, we have given our robot two essential variables, symmetrical in their range of values and dynamics, that need to be kept within permissible limits—lower limit of 0, upper limit of 100—for the robot to remain viable, i.e. survive: *energy* (replenished by consuming ‘food’) and *hydration* (replenished by consuming ‘drink’). They are controlled homeostatically, mostly through interaction with the world when behaviors are executed. They have an ideal value or setpoint (in this case coinciding with the upper limit) and the homeostatic control seeks to bring their actual value as close as possible to the ideal value. The difference between ideal and actual values of the variables gives deficit errors (‘deficits’ for short) that provide the robot with internal—and in this case survival-related—needs that its behavior will try to address. Both deficits increase by 0.1 every 250 ms, so if each deficit starts at 20, the robot will die after 3 min 20 s if it does not succeed in finding and consuming both food and drink before this time.

### 2.2 Perception and actuation

Our robot interacts with its environment constantly. In terms of actuators, it uses its legs to walk, moves its head to visually detect and track objects, and its hand to reach and ‘consume’ food and drink. While interacting, it continuously and asynchronously monitors the environment for percepts potentially relevant to the satisfaction of its needs. We use the standard sensors of Nao, as follows:

- one on-board camera to detect the resources (colored plastic balls) on the grounds of their color and size;
- sonars to detect obstacles;
- contact sensors in the feet to detect collisions;
- gyroscopes to detect inclination of the body and falls.

Food (red) and drink (green) resources (plastic balls) are consumed in discrete chunks (‘bites’), each decreasing the corresponding deficit by 10 units. The resources are not depleted when consumed, and therefore each one is a potentially infinite reservoir.

### 2.3 Motivations

Motivations in animals (including humans) can be defined as ‘inferred internal states postulated to explain the variability of behavioral responses’ (Kandel, Schwartz, & Jessell, 1995, p. 614). In AS, they are modeled as functions that combine the perception of internal deficits and the perception of relevant elements of the environment to provide the robot with urges to action—‘wanting’ to do things—in order to satisfy its needs in the environment in which it is situated. In addition to its longstanding use in the adaptive behavior community (Avila-García & Cañamero, 2005; Cañamero, 1997; Cos, Cañamero, & Hayes, 2010; McFarland & Spier, 1997; Tyrrell, 1993), this notion is broadly used in various disciplines that inform our work, such as animal behavior (Colgan, 1989; Hinde, 1960; Toates, 1995), neuroscience (Kandel et al., 1995; Panksepp, 1998; Pessoa, 2013; Robbins & Everitt, 1999), and psychology (Dai & Sternberg, 2004; Elliot, 2008). In our simple 2RP, each physiological variable has a single motivation associated with it.

As part of the action AS process, motivations are assigned intensity or activation levels that indicate how ‘relevant’ they are, given the robot’s needs and the current external perceptions, as follows

(1) $${\mathrm{motivation}}_{i}={\mathrm{deficit}}_{i}+\underset{\mathrm{perceptual component}}{\underset{︸}{\left({\mathrm{deficit}}_{i}\times \alpha \times {\mathrm{cue}}_{i}\right)}}$$

where $\mathrm{cu}e_{i}$ is the size of the external stimulus, proportional to the radius of the largest corresponding resource detected, or zero when no corresponding resource is detected, and α is a variable (shared across all motivations) that modulates the perceptual component.

As we will see in Section 2.5 and through our experiments, our pleasure mechanism acts on motivations, and hence on the AS process, through modulation of the α parameter. Such modulation of the perception of external stimuli can be thought of as changes in the *attentional effort* (Sarter et al., 2006) or in the *incentive salience* (Berridge & Robinson, 1998) of the stimuli. Therefore, α (and hence, the notion of ‘pleasure’) provides a mechanism to modulate how likely the robot is to interact with the perceived stimuli.

### 2.4 Behaviors

Our architecture has four main or ‘top level’ behaviors: a reflex-like behavior to recover from falls; a ‘return to normal’ behavior that makes the robot adopt a neutral posture when it is not engaged in other activities; and two behavioral subsystems or ‘composite behaviors’ related to the motivations of the robot—*hunger* and *thirst*—and that inherit their intensity or activation levels.^2^

**Figure 2.**
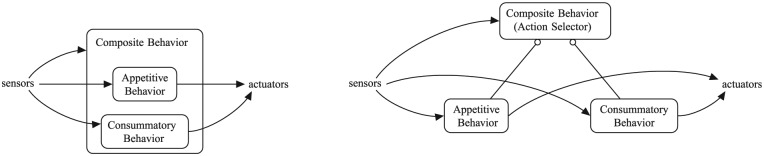
A composite behavior comprising of simpler sub-behaviors—a typical ‘branch’ in our behavior tree. The compact representation, *left*, as used in Figure 1, is expanded into its tree form, *right*, which shows the role of the composite behavior as an action selector.

These motivated behavioral subsystems are composed of a number of smaller behaviors and they are thus ‘action selectors’ themselves, both conceptually and, in our implementation, in an object-oriented sense (Figure 2). The smaller behaviors can, in turn, be composed of yet smaller behaviors, and thus can also be ‘action selectors’. These smaller behaviors can be of two types, as shown in Figure 1—consummatory or appetitive—following the traditional distinction in ethology, neuroscience and AS (Blumberg, 1994; Hinde, 1953; Maes, 1991; McFarland & Spier, 1997; Robbins & Everitt, 1999; Tyrrell, 1993). Consummatory behaviors are goal-achieving and need the presence of a specific incentive stimulus to be executed. Appetitive behaviors are goal-directed search for (or avoidance of) a particular incentive stimulus. In addition to modifying the external environment, the execution of a consummatory behavior has an impact on the level of specific physiological variables; therefore, they are a mechanism to keep the physiological variables viable.

As an example, the *hunger* behavioral subsystem consists of two immediate sub-behaviors: the consummatory *eat food* and the appetitive *seek food*. The latter is in turn composed of four sub-behaviors.

- *Wander*: to walk around the arena when no resource has been detected. This is composed of four sub-behaviors and the interaction of these behaviors, which depend on input from the sensors (e.g. sonars and foot sensors) as well as some random elements, determines the path of the robot.
- *Visual search*: to move the head from side to side. It is continually active, but it is ‘interrupted’ when a food object is detected (when the gaze behavior, which also uses the head, becomes active).
- *Gaze*: at a resource, to turn the head so that the detected food/drink resource that is largest in the camera’s field of view (typically the nearest resource) falls in the center of the visual field.
- *Approach*: to walk towards whatever the robot is facing, turning the body in the appropriate direction. Whenever the approach behavior is active, the gaze behavior will also be active, and the combination of these two behaviors will result in the robot walking towards resources that are detected by the camera.

Each behavior has an activation threshold that determines the level of activation that must be reached for the behavior to be executable, following a process described in Section 2.6. As previously mentioned, multiple behaviors can be executed simultaneously if they do not use the same actuator in a way that makes their simultaneous execution incompatible.

### 2.5 Modeling pleasure

In the experiments presented in this paper, we have used three ‘types’ of pleasure, all of which act on the perceptual element of motivations (the α parameter in equation (1)), although in different contexts.

1. Pleasure modeled by a hormone released *as a function of the satisfaction of homeostatic needs*, building on Cañamero (1997) and Lewis, Hiolle, and Cañamero (2014), that then decays to a background level. This is the model that we describe in this section, and which will be referred to as ‘*Modulated α*’ (Moα) in the experiments (Sections 4 to 6).
2. Different fixed values of ‘hormone’ (which we will refer to as ‘fixed values of α’ in the experiments) were used as *control conditions* in Experiments 1 and 2 (Sections 4 and 5) to compare with the previously mentioned Moα. Such fixed values can be thought of as background levels of hormones unaffected by interactions with the environment, as hormone-releasing chemicals (e.g. drugs) artificially added into the system, or as pathological conditions.
3. Additional hormone (a constant amount) is released *linked to the execution* of consummatory behaviors of ‘eating’ or ‘drinking’ in Experiment 3 (Section 6). This additional release is unrelated to the satisfaction of needs, and corresponds to the *purely hedonic* pleasure mentioned in Section 1. We will refer to this as ‘additional release’ or additional pleasure in the experiments (Section 6).

The rest of this section refers exclusively to the first type of pleasure above. In this context, instantaneous changes in the internal homeostatic variables (mathematically, their first derivative) can be thought of as indicators of the current interactions of the agent with the environment. Our hormonal system reflects improvements (thought of as pleasure) or deterioration (thought of as displeasure) in the interaction with the environment, which we model using the second derivative of the deficit of each homeostatic variable. For the sake of making our investigation incremental, in these experiments we only take into consideration pleasure, i.e. improvements in the interaction with the environment. Since we want our hormone to be released when there is a change resulting in a ‘better’ interaction with the environment, we link hormone release to the *negative second derivative* of any of the homeostatic deficits.^3^ For example, if the robot starts consuming food, the energy deficit decreases (resulting in a negative first derivative); since it was previously not consuming food, the second derivative of the energy deficit is also negative, and hence pleasure hormone is released.

Specifically, our model is as follows. On a 500 ms cycle (empirically determined), we store a history of each deficit’s recent past values $\left[d_{t},d_{t-0.5},d_{t-1}\right]$ where *t* is the time in seconds. This allows us to calculate ‘first derivatives’ as

$${d^{\prime }}_{t}=(d_{t}-d_{t-0.5})/0.5$$

and ‘second derivatives’ as

$${d^{\prime \prime }}_{\;t}=({d^{\prime }}_{t}-{d^{\prime }}_{t-0.5})/0.5$$

If the second derivative is negative, then the level of the hormone is increased by

(2) $$\Delta^{\;\;\;+}\;\;h=-d_{t^{\prime \prime }}\times s$$

where *s* is a scaling parameter. Because our resource items reduce their respective homeostatic deficits by 10, the value of the $d_{t}\prime \prime$ is approximately −40 after each ‘bite’, which means s=0.4 results in 16 units of hormone being released.

The decay of the hormone to a background level is implemented by updating the hormone level *h* once per second according to the rule

(3) $$\Delta^{-}h=-(h-b)\times k$$

where *b* is the background level and *k* the decay rate. The background level corresponds to a state of equilibrium between the release of the hormone and its natural decay. We use values b=10 and k=0.19, giving the decay a half-life of 3.3s relative to the background level. These values were determined empirically to be meaningful for the interaction dynamics in our environment, so that the presence of the hormone is not so fleeting that it has little chance of affecting the behavior, and not so long-lasting that it affects the interaction long after the initial release.

**Figure 3.**
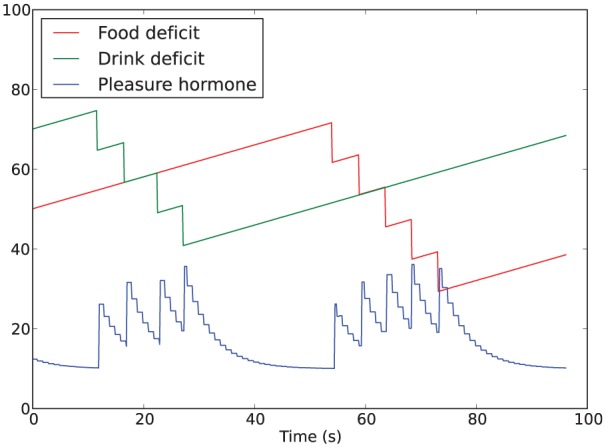
Example of the hormone being released with decreases in the homeostatic deficits and then decaying to the background level.

In summary, the dynamics of the hormone, illustrated in Figure 3, depends on three factors:

- its background level, *b*;
- its decay rate, *k*;
- the relationship between homeostatic variables and hormone release—a linear scaling by *s* of the second derivative.

In those experimental conditions where the hormone level affects α (the Moα condition in Sections 4.2 and 5.2, and all the conditions in Section 6.2), these three factors will therefore also affect the behavior of the robot.

### 2.6 Action selection process

The AS process involves various elements running on different time scales. Each AS ‘cycle’ is thus not a sequential loop, but a number of asynchronous loops running in parallel. The main elements can be grouped as follows.

1. The robot is continuously ‘monitoring’ its environment for percepts that might be relevant to need satisfaction and its interaction with the world. Different sampling rates are used for different sensors as appropriate.
2. The physiology (i.e. the values of the internal variables and the pleasure hormone) is updated.
3. Motivational intensities are calculated and passed on to the top-level behavioral subsystems.
4. A behavior selection cycle selects the behavior(s) to be executed every 125 ms, i.e. 8 times per second. This cycle is a sequential loop that can be summarized as follows. Each behavior, starting with the top-level behavioral subsystems:
  - gets the activation levels of its sub-behaviors;
  - sorts the sub-behaviors according to their activation levels, highest to lowest;
  - for each sub-behavior in its sorted list, this behavior (in order):
    – checks if it is inactive (i.e. if its activation level is below its activation threshold). If so, it skips to the next sub-behavior; if not:
    – checks if any of the actuators needed for the sub-behavior are already in use by ‘extended behaviors’.^4^ If so, it skips to the next sub-behavior; if not:
    – checks if any of the actuators needed for the sub-behavior have already been used by behaviors already executed in this cycle. If so, it skips to the next sub-behavior; if not:
    – selects the sub-behavior (which may itself be an action-selector), which is executed. If this sub-behavior is an extended behavior, then it tags those actuators it is using as ‘in-use’.^5^ The extended behavior then spawns a separate thread in which the main part of its execution occurs.
    – after it has been executed, this sub-behavior returns the actuators that it used to the parent behavior, which adds them to the list of actuators already used in this cycle.

## 3 Experiments’ method and metrics

### 3.1 Method

To facilitate systematic analysis of results, we decided to test our model of pleasure using a 2RP (Spier and McFarland, 1997). Even this simple problem can give rise to a number of variations with potential consequences for the viability and decision-making behavior of the robot. For this study, we have manipulated the following: the availability of resources (easy/difficult access), their (symmetric or asymmetric) distribution, and how the release of pleasure relates to their consumption—either to their ‘nutritional value’ or simply to the act of consuming.

The arena used to design the environments of all our experiments is a 2 m×2 m area bounded by wooden boards that, at 40 cm high, are easily detected by the robot’s sonars. Red (‘food’) and green (‘drink’) balls, used as resources, were placed at the top of the walls, where they are easily visible to the robot’s camera; in one of the experiments some resources were also attached to a box placed in the middle of the arena. The number and distribution of the green and red resources varied according to the conditions tested in each experiment, as shown in Figures 5, 10 and 15. To make the robot’s color-based object recognition more reliable, a sheet of white paper was attached to the wall immediately below each resource, to make them stand out more.

### 3.2 Metrics

We use the following metrics to assess the robot’s performance and to characterize relevant aspects of its behavior.

#### 3.2.1 Viability indicators: Comfort and discomfort

We use indicators of performance based on the notions of viability and ‘wellbeing’ (Ashby, 1960; Avila-García & Cañamero, 2004) to assess different aspects of how the viability of the physiology is maintained in the interactions of the robot with its environment.

Unlike in previous work, e.g. (Avila-García and Cañamero 2004; and Cos et al., 2013), which builds on the notion of ‘comfort’, here we use the converse notion of ‘discomfort’ that increases and decreases following the deficits (rather than inversely to them), and hence their link between the metrics and the deficits is intuitively easier to see. In addition to using the arithmetic mean of the deficits at time *t* and the variance of the deficits at time *t* as measures of ‘overall discomfort’, we introduce a measure of discomfort based on the *geometric* mean.^6^ We define arithmetic and geometric discomfort (and comfort) as:

- The *arithmetic discomfort* at time *t*, $D_{A}(t)$, is defined as the (arithmetic) mean of the deficits, $d_{i}(t)$, i.e. $D_{A}(t)=\frac{1}{n}\sum_{i}d_{i}(t)$ where *n* is the number of homeostatic variables. The *arithmetic comfort* is then defined as $C_{A}(t)=100-D_{A}(t)$, or equivalently the (arithmetic) mean of the values $100-d_{i}(t)$. This notion of ‘arithmetic comfort’ is identical to the notion of ‘overall comfort’ defined in (Avila-García and Cañamero, 2004).
- The *geometric comfort* at time *t*, $C_{G}(t)$, is defined as the geometric mean of the values $100-d_{i}(t)$, i.e. $C_{G}(t)=\sqrt[{n}]{\Pi_{i}(100-d_{i}(t))}$. The *geometric discomfort* is then defined as $D_{G}(t)=100-C_{G}(t)$.

We prefer to use the geometric (rather than the arithmetic) discomfort, as it has the advantage that the discomfort is at its maximum value (100) if and only if the agent is dead, whereas the arithmetic discomfort would make possible the counter-intuitive situation where a dead agent could have less discomfort than a living agent. In addition, from the well-known relationships of the geometric and arithmetic means, we see that $D_{G}(t)\geq D_{A}(t)$ and these notions of discomfort are equal if and only if the deficits are equal; in other words, the geometric discomfort is closer to the largest deficit (the most pressing homeostatic need) than the arithmetic discomfort is.

#### 3.2.2 Behavioral metrics: Persistence and opportunism

These metrics take into account two of the key problems that AS architectures should be able to tackle: persistence and opportunism (Maes, 1995; Tyrrell, 1993). Generally speaking, in the AS literature, *persistence* is related to the ability of an agent to continue working towards its most relevant ‘goals’, while *opportunism* has to do with its ability to take advantage of relevant opportunities and contingencies offered by the environment. For an autonomous robot that has to satisfy multiple conflicting survival-related needs, it is crucial not only to choose behaviors that do so in a timely fashion, but also to persist in their execution for long enough to guarantee sufficient satisfaction. Persistence is important to avoid what is known as the ‘dithering’ problem, which occurs when a robot keeps switching between trying to satisfy two needs without satisfying any of them enough to guarantee survival. Closely related to persistence, opportunism is the consumption of a resource that might not be needed at present but is available now and might not be available later. The degree to which a robot should show persistence and opportunism depends on multiple factors; we could generally say that persistence leads to a more ‘conservative’ AS behavior and opportunism to a more ‘risky’ one. In previous work (Avila-García & Cañamero, 2004; Lewis et al., 2014), we showed that persistence and opportunism can also become negative when done in excess, and proposed initial mechanisms inspired from emotions in natural systems and based on hormonal modulation of the perception of external stimuli (the resources), to address these problems.

We first consider definitions of persistence and opportunism that we call ‘persistent’ and ‘opportunistic’*periods ofconsumption* to distinguish them from our later definitions of *periods of* *attempted* persistence and opportunism.

**Figure 4.**
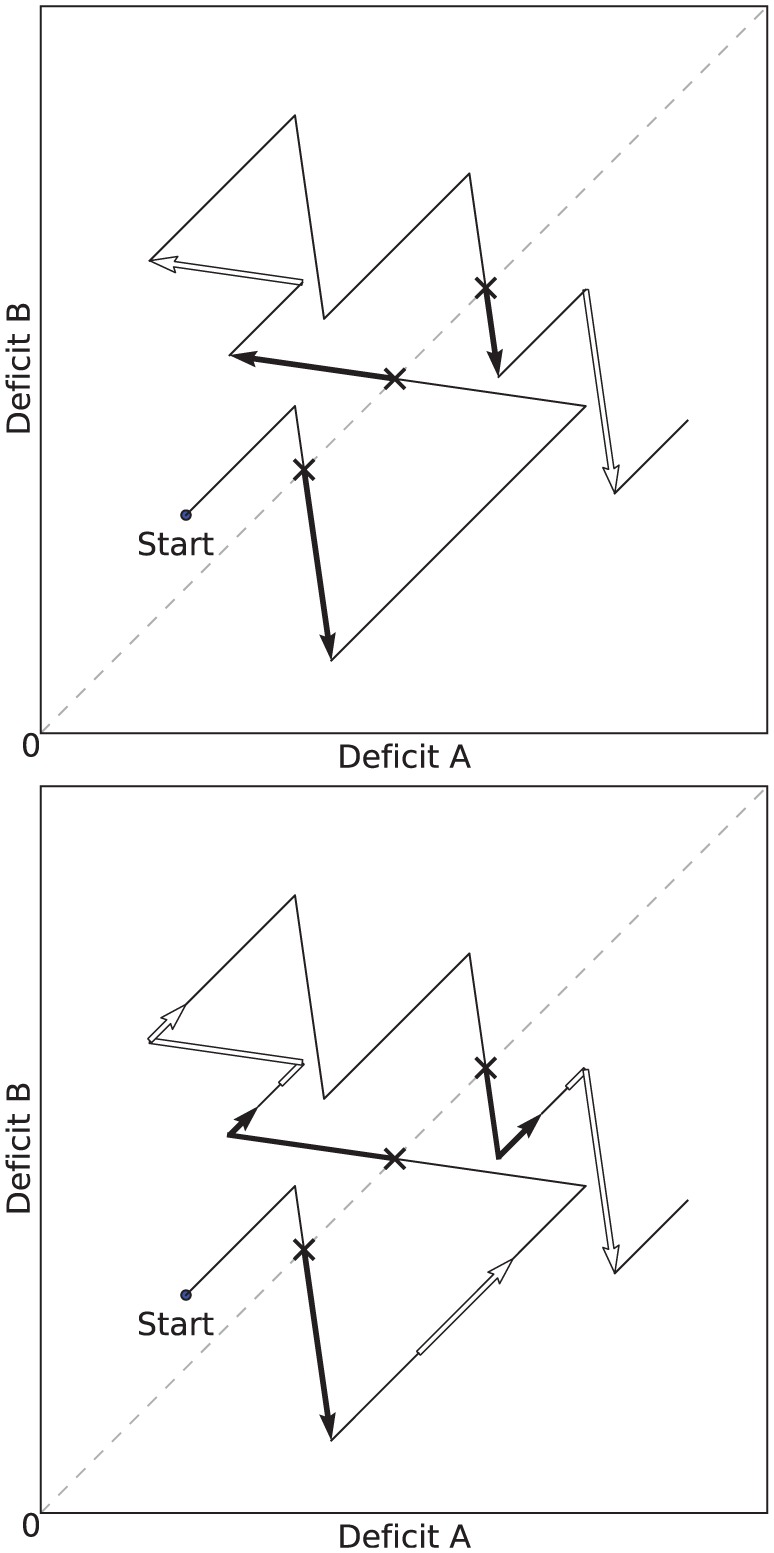
Example plots in a two-resource ‘physiological’ or ‘deficit’ space. The arrows mark periods of persistent/opportunistic consumption (*top*), and attempted persistence/opportunism (*bottom*). Both plots show the same evolution of the deficits. From the starting point, the deficits increase as the robot is not consuming any resource. Shortly after starting, the robot begins to consume a resource which decreases Deficit B (the most pressing homeostatic need at this point), while Deficit A continues to increase. During consumption, a first crossover point is reached as both deficits reach the same level (crossover points are marked by a cross), and subsequently a first period of persistence starts. *Top*: Periods of *persistent consumption* are shown as filled arrows, and periods of *opportunistic consumption* by unfilled arrows. *Bottom*: Periods of *attempted persistence* are shown as filled arrows, and periods of *attempted opportunism* by unfilled arrows. In this plot we see that during the long period where both deficits were increasing, there is a period of attempted opportunism—the agent attempted to reduce Deficit B, but it failed, leading to a costly increase in both deficits, and delaying the consumption of a resource to reduce Deficit A.

- We define a period of *persistent consumption* as a period where the consumption of a resource that had started when its corresponding deficit was the largest, has continued beyond the crossover point in the ‘deficit’ or ‘physiological’ space (Figure 4). The crossover point is the point at which the largest deficit falls below another deficit and is no longer the largest.
- We define a period of *opportunistic consumption* as a period of consumption that started when the corresponding deficit was not the largest or equal to the largest.

These definitions of persistence and opportunism only take into account occurrences when resources are successfully consumed. However, one reason for considering persistence and opportunism is that they should occur in a way that balances their possible benefits (consumption of resources) against their costs (time that could otherwise be used seeking resources to satisfy more pressing deficits). Our previous definitions of persistent and opportunistic consumption only include periods where the benefits (successful consumption) are gained, and not periods involving cost (time wasted attempting but failing to access a resource). To take into account these latter periods, we introduce the notions of *attempted* persistence and opportunism (Figure 4, *bottom*).

- We define a period of *attempted opportunism* to be one in which all of the following hold:
  1. Any of the (consummatory or appetitive) behaviors associated with a resource *R* are being executed.
  2. The deficit associated with resource *R* is not the largest (or equal largest) of the deficits.
  3. Immediately prior to the period, none of the behaviors from (1) were active.
- We similarly define a period of *attempted persistence* to be one in which (1) and (2) above hold, and in addition:
  3. Immediately prior to the period, at least one of the behaviors from (1) was active, and the associated deficit was the largest (or equal largest) of the deficits.

## 4 Experiment 1: Comfortable environment

The first set of experiments were carried out in a simple, unchallenging ‘baseline’ environment in which resources are plentiful, equally distributed and easily accessible. The robot should be able to satisfy its needs so as to normally survive for the entire duration of the runs. In this experiment we are not assessing the role of pleasure in terms of its utility for survival, but rather whether pleasure makes a difference in terms of (a) how viability or wellbeing is maintained and (b) the type of behavior exhibited by the robot.

### 4.1 Experimental setup

**Figure 5.**
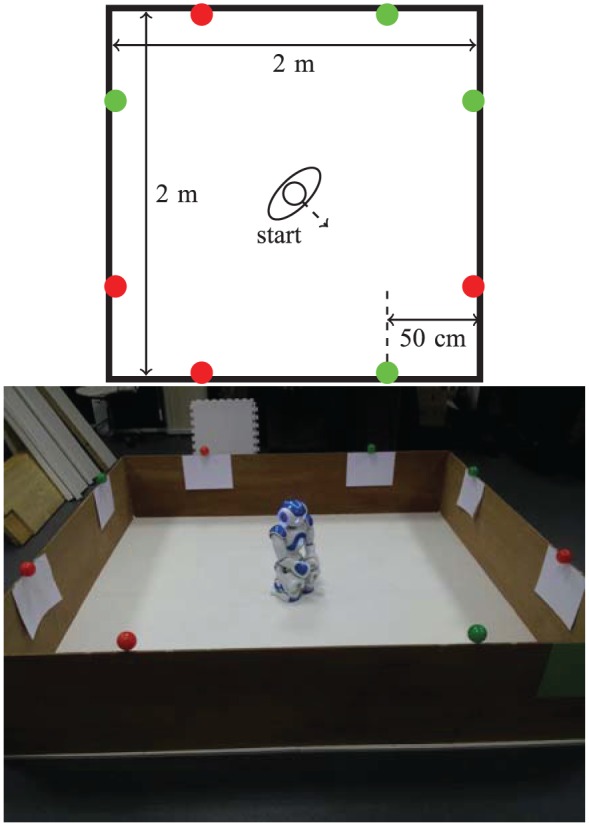
A diagram and photo of the arena used for Experiment 1, showing the robot’s starting position and the location of the resources.

Four red (‘food’) resources and four green (‘drink’) resources are fixed to the top of the walls of the arena, where they are easily visible to the robot’s camera, as shown in Figure 5. This environment is symmetric in the following ways.

1. The number of items of each resource is identical.
2. The ‘nutritional value’ of both resources is identical.
3. The metabolism and physiological changes (e.g. rates of growth and satiation of the deficits, ideal value and fatal limits of the variables) associated with both resources are identical.
4. The conditions tested (Section 4.2) were identical for each resource. More specifically, the values of α associated with the consumption of both resources are identical for the fixed α conditions, and the amount of pleasure hormone released as a result of consuming the resources in the Moα condition is also the same for both resources.
5. The resources are symmetrically distributed in the environment (Figure 5) as follows: reflection in one diagonal (the diagonal along which the robot is facing at the beginning of each run) would result in the resources swapping (complementary symmetry: red ↔ green); reflection in the other diagonal would leave the resources unchanged (mirror symmetry: red → red, green → green).
6. The starting position of the robot (in the middle of the arena) affords an equal view of the two resources.

### 4.2 Experimental conditions tested

We compared four different α conditions—three different fixed values and one in which its value was modulated by a pleasure hormone released as described in Section 2.5, as follows.

- Condition 1, ‘Low Fixed α’ (LFα): α=0.1.
- Condition 2, ‘Medium Fixed α’ (MFα): α=0.35.
- Condition 3, ‘High Fixed α’ (HFα): α=0.6.
- Condition 4, ‘Modulated α’ (Moα): α is proportional to the level of the pleasure hormone, specifically

(4) α=0.01×hormonelevel

The particular value 0.01 was chosen so that α would have a range of the same order as the fixed values in Conditions 1–3.

The specific LFα value of α=0.1 and the value of the parameters setting the background level of pleasure (b=10 in equation (3) and the above scaling factor of 0.01) in the Moα condition were chosen so that LFα and the background level of pleasure in the Moα condition would be equal.

We conducted a total of 40 runs—10 runs for each condition. The order of runs was randomized, with runs in each condition spread across the set of runs. This was done by generating 10 random orderings of the numbers 1 to 4. All runs were done in the same artificial light conditions. To avoid potential differences in the functioning of sensors and actuators, breaks (of varying length depending on practicalities, but of at least 10 min) were taken between runs to recharge the battery and to allow the joint motors to cool down. On each run, the robot started at the center of the arena, facing the same corner. Each run lasted either until the robot ‘died’ (one deficit passed the fatal limit), or until 6 min had passed. The data recorded during each run comprise: the values of the deficits, the motivations, the hormone level, the resources detected, and the currently active behaviors.

### 4.3 Results

**Figure 6.**
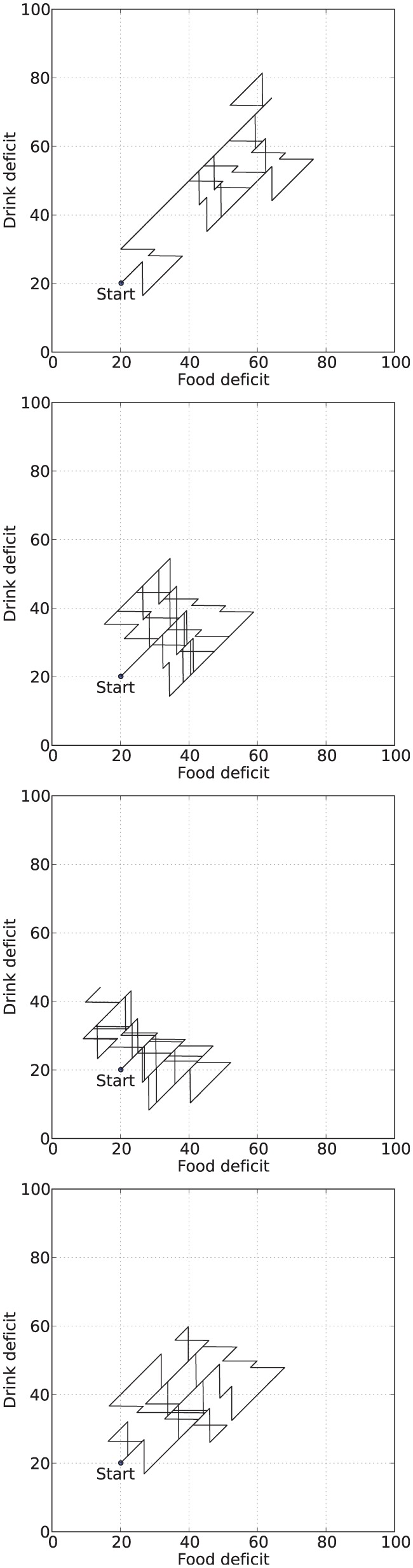
Example deficit–space plots from Experiment 1. From top to bottom: α=0.1, α=0.35, α=0.6, Modulated α.

**Figure 7.**
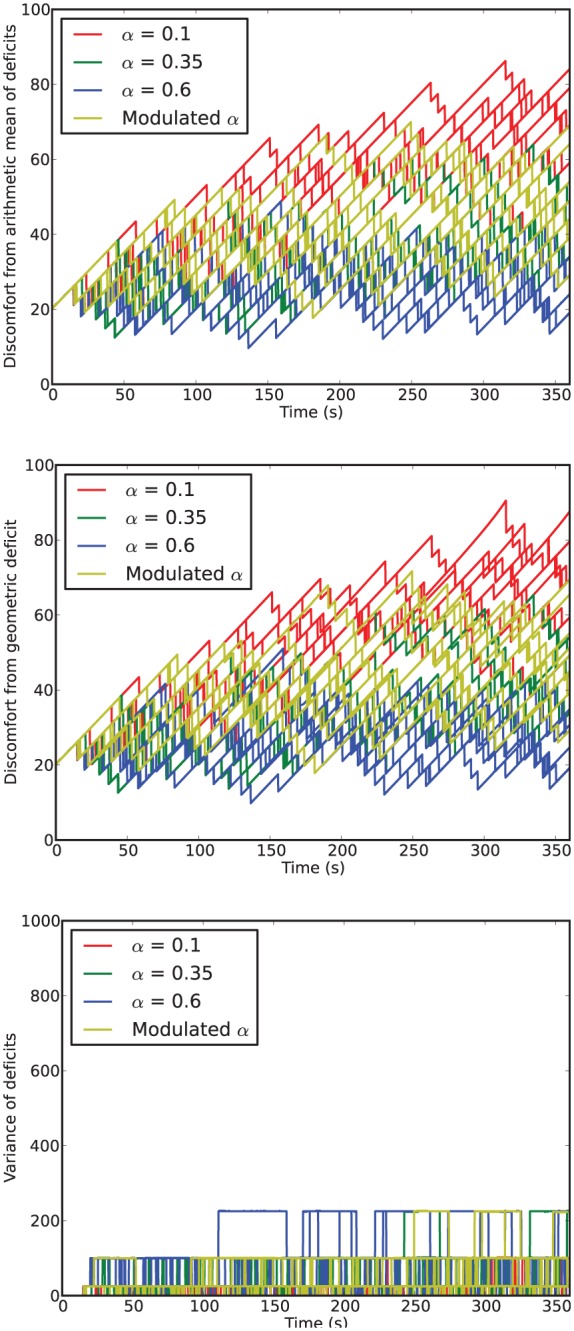
Time series of discomfort metrics from Experiment 1.

**Figure 8.**
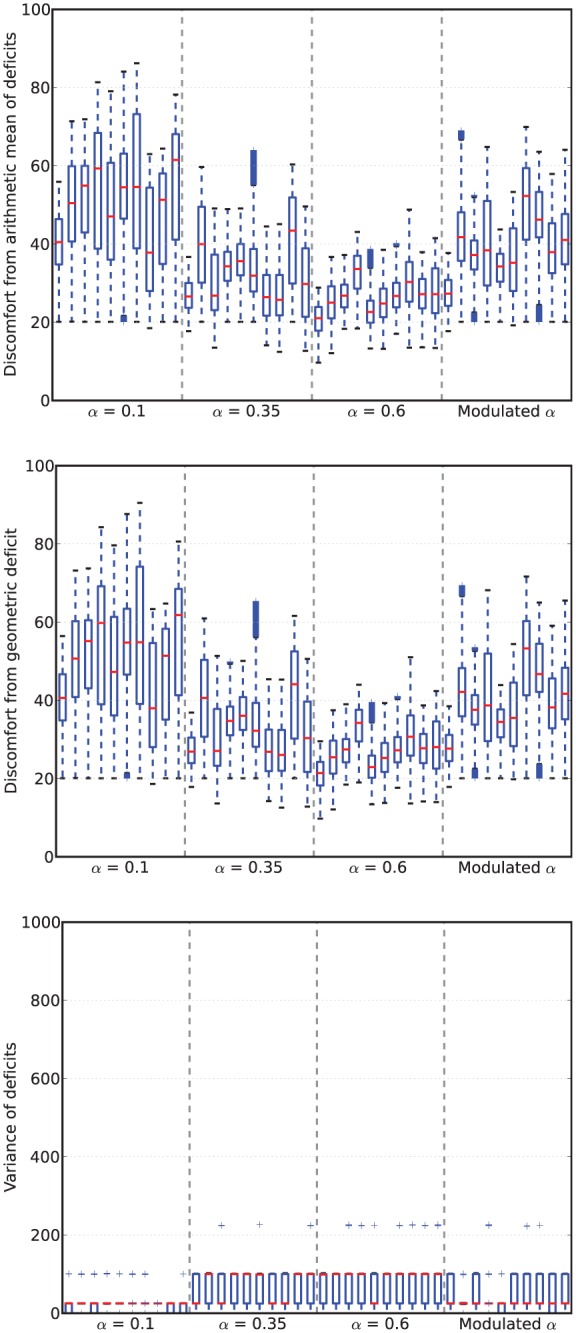
Viability metrics from runs in Experiment 1. In the box-and-whisker plots, the red lines show the medians, the boxes contain the middle quartiles of the data and thus give an indication of the distribution of the deficits over time.

Figure 6 shows example deficit–space plots from single runs under each condition. As we can see, increasing the value of α results in the homeostatic variables remaining closer to their ideal value (zero deficit). Figure 7 shows the discomfort metrics for each run plotted against time and Figure 8 the distribution of the performance metrics for each run.

In this environment, the robot survived the full six minutes every run, confirming that it is not a challenging environment. However, there are clear differences between the four conditions.

For fixed values of α—Conditions 1 (LFα), 2 (MFα) and 3 (HFα)—the discomfort decreased (means of the geometric discomfort over all the runs are 49.8, 33.3, 27.1 for Conditions 1, 2 and 3, respectively) and the mean variance increased (means are respectively 28.0, 59.3, 71.2 for Conditions 1, 2 and 3) with increasing values of α. Therefore, in this environment and within the range of values that we have tested, increasing α as much possible is best for the robot in terms of discomfort.^7^

**Figure 9.**
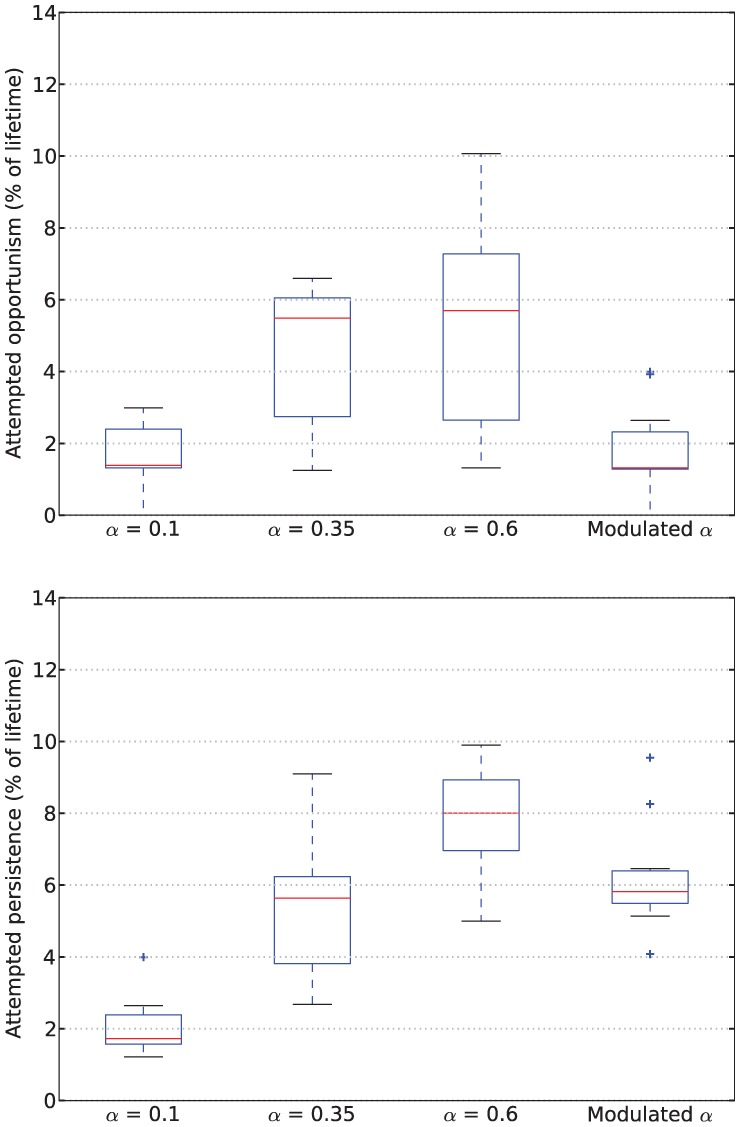
Rates of attempted opportunism (*top*) and attempted persistence (*bottom*) from Experiment 1.

Examining the behavior of the robot in terms of persistence and opportunism, we see in Figure 9 that for fixed values of α (Conditions 1, 2 and 3), the amount of attempted persistence increases with α. Opportunism also increases from low to medium α (Conditions 1, LFα with α=0.1, and 2, MFα with α=0.35), but it is not clear whether there is a change between Condition 2 (MFα, α=0.35) and Condition 3 (HFα, α=0.6).

Turning to the results for the Modulated α (Moα, Condition 4), we see that its geometric discomfort metric (mean over all the runs 39.7) and the variance of its deficits (mean 47.0) both tend to lie between those for Conditions LFα (α=0.1) and MFα (α=0.35).

However, looking at persistence and opportunism, we see in Figure 9 a very different picture: it shows that opportunism has rates similar to those of α=0.1 (Condition 1, LFα) and persistence has rates similar to those of α=0.35 (Condition 2, MFα). The robot’s behavior under the Moα condition thus differs from all of those with fixed values of α. This means that the strategy that the robot uses to achieve a comparable level of performance in terms of our viability metrics is measurably different when considered in terms of our behavior metrics—persistence and opportunism.

Comparing the arithmetic and geometric discomforts (Figure 8, *top* and *center*), the choice of either metric makes little difference to the overall picture: low and high levels of discomfort remain low and high whichever definition is used. At high levels of discomfort (observed in the LFα condition) the values of the geometric discomfort are larger, more accurately conveying the closeness of the deficits to the fatal limit, therefore supporting our preference for the geometric over the arithmetic discomfort.

## 5 Experiment 2: Difficult access to resources

In the benign environment of Experiment 1, there was little cost for attending to the current need until satiated, since both types of resources were easily perceived and accessed. In this second experiment, we introduce a cost associated with the access to resources, more precisely with the ability of the robot to perceive the resources.

### 5.1 Experimental setup

**Figure 10.**
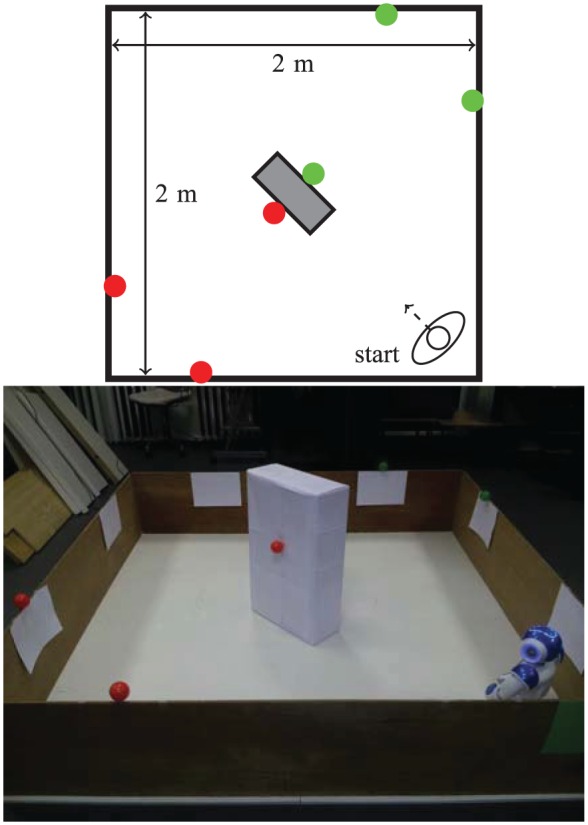
A diagram and photo of the arena used for Experiment 2.

In this environment (Figure 10), the robot might need to wander around the environment before it can detect some of the resources. To achieve this, we have:

- placed an obstacle (white cardboard box) in the center to make it more difficult for the robot to detect resources on the other side of the arena;
- placed resources so that one area contains only food resources and the other only drink resources.

To consume the resources, the robot needs to move from one section of the arena to the other. In this case, being ‘distracted’ by attempted opportunism would prevent it from doing this efficiently and hence would have a negative impact on viability. On the other hand, the robot still needs to show appropriate levels of persistence in order to consume enough of a resource to counterbalance the growth of the deficit during the exploratory journey from one section of the arena to the other and then back again.

This environment is symmetric in terms of the properties listed in Section 4.1 for the first experiment, although not exactly in the same way. Both environments are identical regarding properties (1)–(4), but the environment in the present experiment differs regarding properties (5) and (6) as follows:

5. Distribution of resources: reflection in one diagonal (along which the robot is facing at the beginning of each run) would result in the resources swapping (complementary symmetry: red ↔ green); reflection in the other diagonal would leave the resources unchanged (mirror symmetry: red → red, green → green).
6. The starting position of the robot (a corner of the arena) affords an equal view of the two areas, and hence resources.

### 5.2 Experimental conditions tested

We ran the robot in the same Low, Medium and High fixed α conditions (α=0.1, 0.35, 0.6) and the Modulated α condition used in Experiment 1 (see Section 4.2), in a randomized order and in the same artificial light conditions. Also, as in Experiment 1, the runs lasted either until the robot died or until six minutes had passed. The battery was charged regularly, and time was allowed between runs to allow the joints to cool. The data recorded during each run comprise: the values of the deficits, the motivations, the hormone level, the resources detected, and the currently active behaviors.

### 5.3 Results

**Figure 11.**
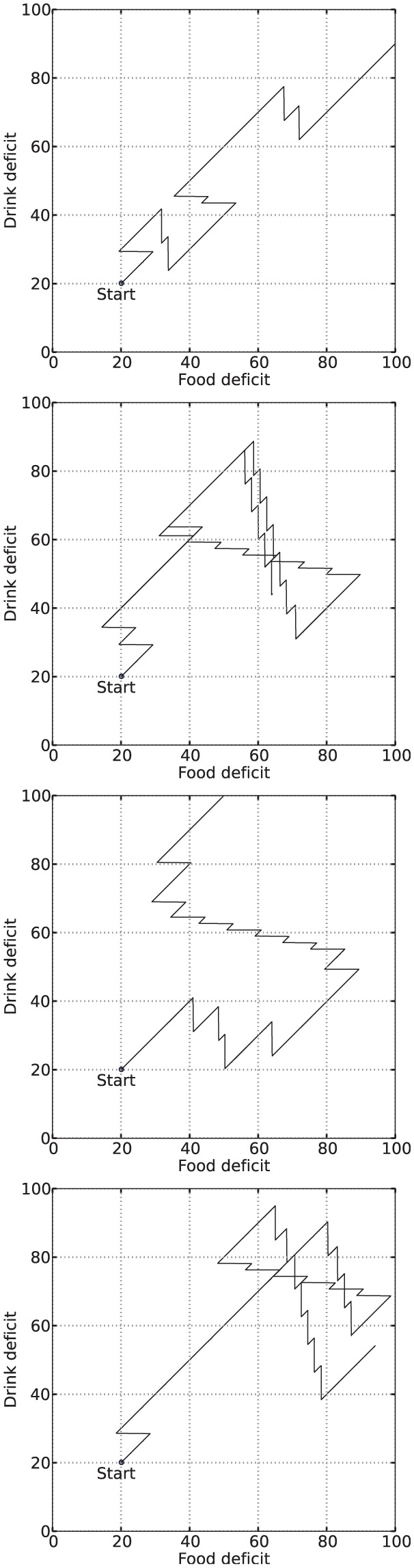
Example deficit–space plots from Experiment 2; from top to bottom: sample runs with LFα (α=0.1), MFα (α=0.35), HFα (α=0.6), and Modulated α (Moα). The LFα condition almost invariably leads to death as the robot does not persist enough in consumption of resources; MFα shows increased persistence and hence better viability; in runs with HFα, the robot dies more often than in those with MFα; in the sample run with Moα shown here, the robot survives for the total maximum time of 6 min, but comes close to the fatal limit, and in half of the Moα runs the robot died.

**Figure 12.**
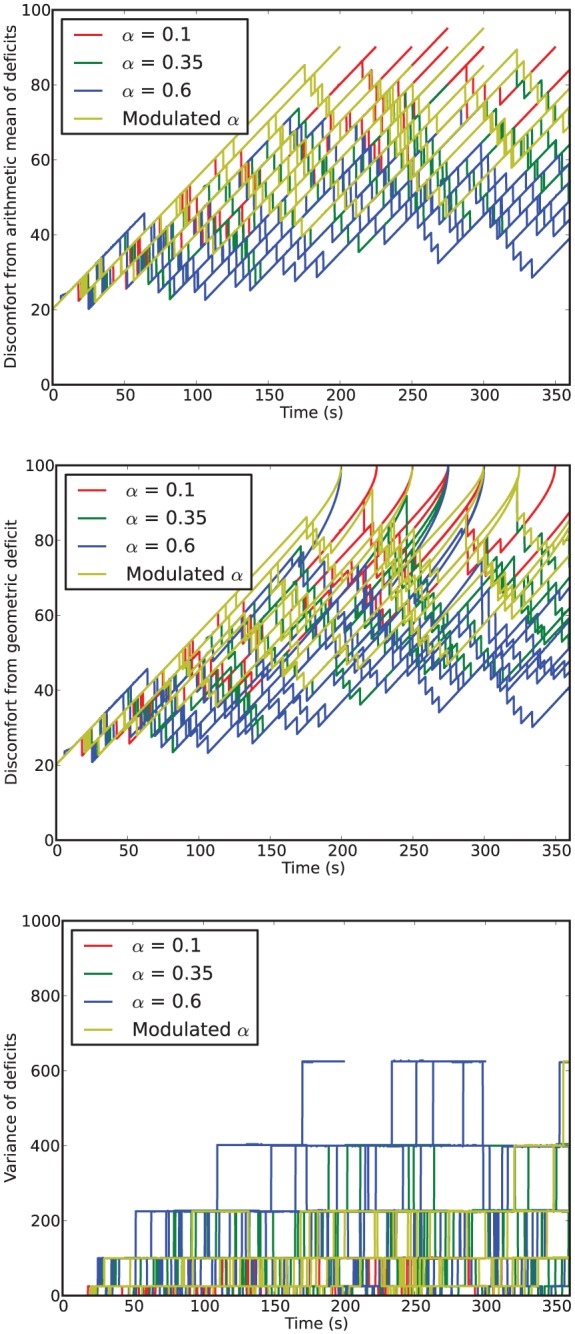
Time series of discomfort metrics from Experiment 2.

**Figure 13.**
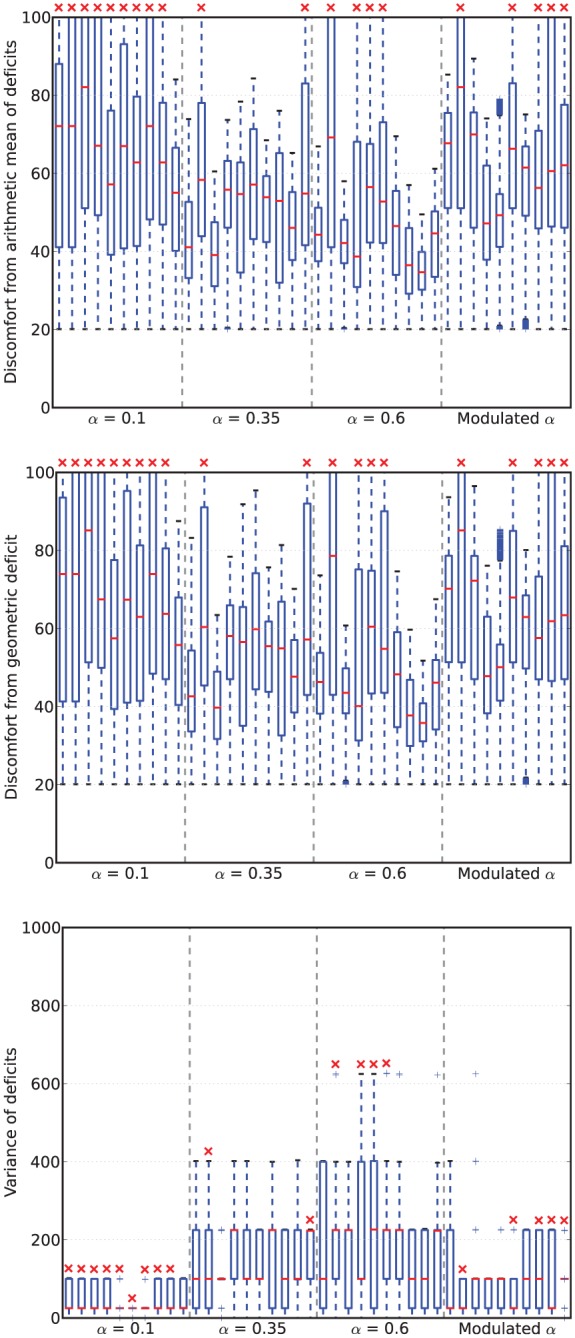
Viability metrics from runs in Experiment 2. In the box-and-whisker plots, the red lines show the medians, the boxes extend to the upper and lower quartiles of the data. The red crosses indicate those runs in which the robot died.

**Figure 14.**
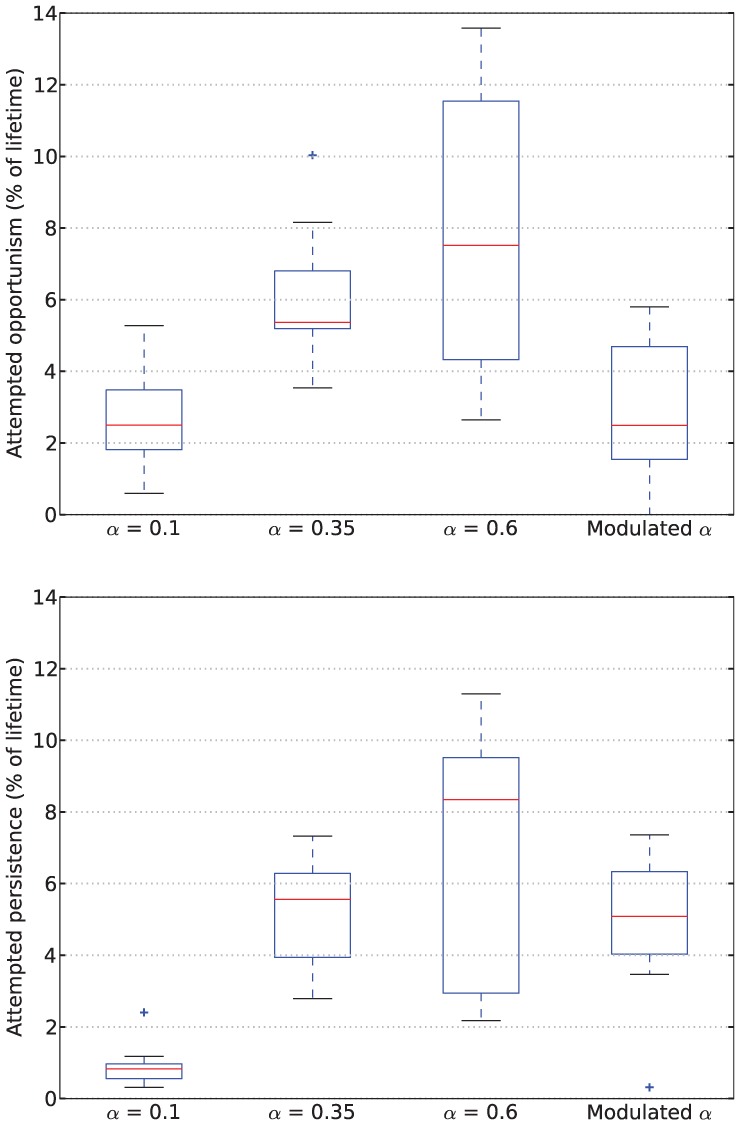
Rates of attempted opportunism (*top*) and persistence (*bottom*) from Experiment 2.

Figure 11 shows deficit–space plots from example runs under each condition. Figure 12 shows the discomfort metrics for each run plotted against time and Figure 13 the distribution of the performance metrics for each run. Note that in cases where the robot died, when calculating mean discomforts over the 6-min run, we set the discomfort of a dead robot to be 100—the maximum value—so that the metrics took account of the death of the robot. Figure 14 shows rates of opportunism and persistence.

In terms of survival, this environment is clearly more challenging than that used in Experiment 1: all but one of the LFα runs resulted in the death of the robot, and two of the MFα, four of the HFα and five of the Moα runs resulted in death. It is noteworthy that the highest α does not result in the lowest number of fatalities—unlike in the environment used in Experiment 1, this environment punishes a high α.

However, the number of deaths does not match the pattern observed in the discomfort. Here, the mean geometric discomforts for increasing α are 65.7, 52.8, and 50.6 for the LFα, MFα, and HFα conditions, respectively. For the Moα condition, the mean of the geometric discomforts over all the runs is 61.9, i.e. between the values for the LFα and MFα conditions.

Looking at the variance, we see that it increases with increasing fixed α values: mean values over the lifetime of the robot are 44.9, 146.9, and 192.4 for the LFα, MFα, and HFα conditions, respectively. The variance for the Moα condition is 89.1, between the values for the LFα and MFα conditions.

## 6 Experiment 3: Introducing asymmetry

In the two previous experiments, we maintained symmetry between the two resources on a number of key features, as mentioned in Sections 4 and 5. In this third experiment, we introduce asymmetry both in the environment and in the role of pleasure.

### 6.1 Experimental setup

**Figure 15.**
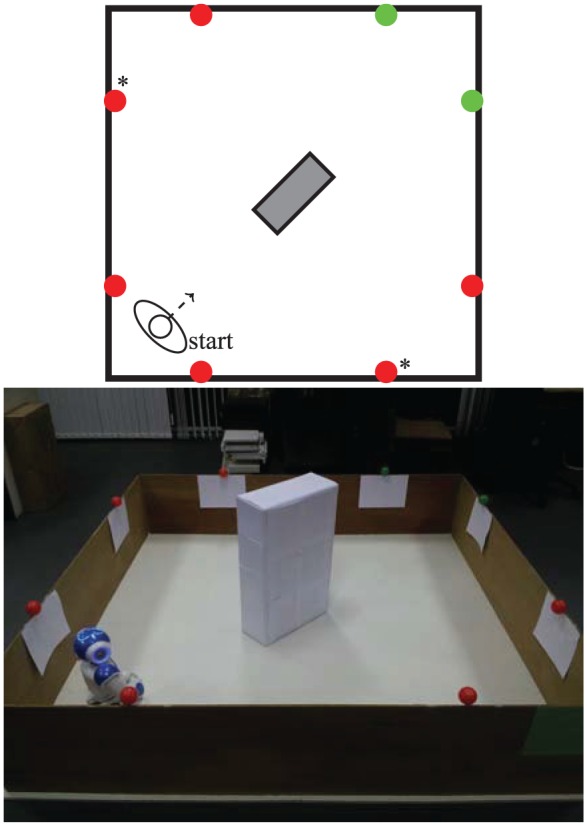
A diagram and photo of the arena for Experiment 3, showing the asymmetric resource layout. To achieve this layout, we modified the environment used in Experiment 1 by replacing two of the food resources with drink resources (marked with asterisks in the diagram). The barrier (the box in the center of the arena) used in Experiment 2 was placed in the center of the arena, so that when seeking a drink resource the robot would sometimes go the long way around. Without the box, the robot would simply go directly to the drink resources, and this would mean that, although there were fewer of them, there would have been little decrease in their availability, making the environment too ‘easy’.

Asymmetry is introduced in the environment (Figure 15) regarding availability of the two types of resource, making one of them abundant—by using six food resources—and the other scarce—only two drink resources are used, and the presence of the box in the middle of the environment ensures that they cannot be perceived from all the locations in the environment.

For comparison purposes, in two of the conditions tested (Conditions 1 and 2 in Section 6.2) we use a symmetric environment. This environment was identical to the asymmetric environment, except that the food resources marked with an asterisk in Figure 15 were replaced with drink resources. There were thus four of each resource.

One might intuitively hypothesize that the introduction of asymmetry in the number of resources, whilst maintaining the symmetry in the pleasure obtained from both resources, would lead to the case in which pleasure would be maladaptive since it would make the robot consume even more of the abundant resource and neglect the scarce resource. To investigate whether this would be the case, we also introduced asymmetry in the release of pleasure, making one of the two resources more pleasurable than the other. Asymmetry in the role of pleasure is thus produced by changing the amount and the context in which the pleasure hormone was released for each of the resources.

We made the effects of pleasure from *consuming* the two resources asymmetric, giving rise to two conditions: more pleasure associated with the consumption of the abundant resource, and more pleasure associated with the consumption of the scarce resource. To better assess the effects of this ‘extra pleasure’, we decided to decouple it from the nutritional value of the resources and hence from the satisfaction of physiological needs. This separation is also supported by recent results in neuroscience (Tellez et al., 2016).

**Figure 16.**
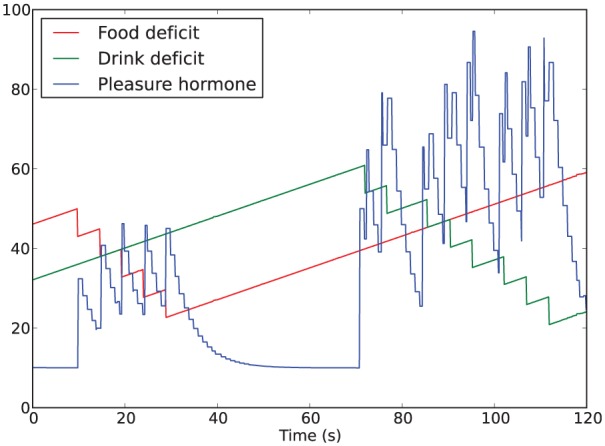
Example of the hormone dynamics from Experiment 3 with hormone also released on execution of the drinking behavior (Condition 4). Consuming drink results in paired spikes showing the two separate releases of hormone, and a larger overall release of hormone.

We have thus introduced a second mechanism for the release of the pleasure hormone on top of the first mechanism linked to the changes in the satisfaction of physiological needs: successful execution of the consummatory behavior of one of the resources (consuming either the abundant or the scarce resource, depending on the condition) results in a release of 40 units of the pleasure hormone, independent of any release due to the regulation of the homeostatic variables (Figure 16). This additional trigger for hormone release can be considered analogous to ‘tasting good’, or generally just ‘liking’—sensory pleasure from just the act of consumption, regardless of the physiological benefit or utility of what is consumed (Cabanac, 2010; Frijda, 2010).

Considering the environmental properties in Section 4.1, the present environment is identical regarding properties (1) and (3), but differs regarding the remaining properties as follows.

2. The ‘nutritional value’ of both resources is identical in all conditions (though these values are different from those in Experiments 1 and 2).
4. The amount of pleasure hormone released as a result of homeostatic changes due to the consumption of resources is the same for both resources. However, in some conditions, there is an additional release of pleasure hormone associated with the consumption of one type of resource, but not the other.
5. The resources are symmetrically or asymmetrically distributed, depending on the condition. In the symmetric conditions the placement and distribution of resources is the same as in Experiment 1. In the asymmetric conditions, mirror symmetry is used in the axis along which the robot is facing at the beginning of each run. However, since we have different numbers of each resource, distribution is asymmetric on the other axis.
6. The starting position of the robot (a corner of the arena) affords an equal view of the two areas, and hence resources.

### 6.2 Experimental conditions tested

In this experiment the fixed α conditions are not used, since we focus on a pleasure-modulated α with the pleasure hormone released in different contexts.

Combining the two sets of criteria—asymmetry in the environment and asymmetry in the role of pleasure—we obtain six conditions. These six theoretical conditions in fact amount to five experimental conditions, since in the symmetric environment adding additional asymmetrical hormone for either of the two resource types would give exactly equivalent conditions. Therefore, we report only the five distinct experimental conditions (summarized in Table 1) as follows.

- Condition 1, baseline: symmetric environment, symmetric pleasure released only from changes in essential variables.
- Condition 2: symmetric environment, asymmetric pleasure. The pleasure hormone is released from essential variables, and there is additional release from consuming drink.
- Condition 3: asymmetric environment, symmetric pleasure released only from changes in essential variables.
- Condition 4: asymmetric environment, asymmetric pleasure. The pleasure hormone is released from essential variables, and there is additional release from consuming drink—the scarce resource.
- Condition 5: asymmetric environment, asymmetric pleasure. The pleasure hormone is released from essential variables, and there is additional release from consuming food—the abundant resource.

**Table 1.** The five conditions tested in the Experiment 3.

	**Symmetric environment**	**Asymmetric environment** (plentiful food, scarce drink)
**Symmetric pleasure**	Condition 1 (baseline)	Condition 3
**Asymmetric pleasure** (additional pleasure from *drink*)	Condition 2	Condition 4
**Asymmetric pleasure** (additional pleasure from *food*)	(same as Condition 2, not reported)	Condition 5

In order that the robot’s actions have a more fine-grained effect on its internal deficits, which will allow us to see any asymmetries more clearly, we have reduced the ‘nutritional value’ of both resources from 10 to 7 units (established empirically). This means that, in Equation (2), d″ for each bite is now −28, and our new choice of s=0.8 results in a release of 21.6 units of hormone. Without these changes in the parameters, asymmetries in the robot’s behavior would still be there, but be harder to measure.

As in Experiments 1 and 2, we conducted 10 runs in each condition, giving a total of 50 runs.

### 6.3 Results

**Figure 17.**
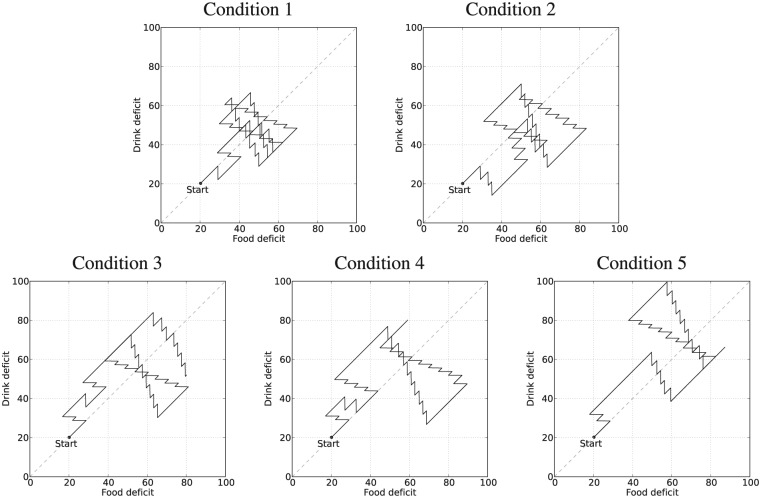
Example deficit–space plots from Experiment 3. Diagonal lines have been added to make the asymmetry more visually clear. **Top row:** the symmetric environment. *Top left*: symmetric pleasure, the balance between the deficits (both in terms of time spent satisfying each need and the location in the physiological space) can be seen in the symmetry of the plot. *Top right*: additional pleasure from drinking results in more persistent drinking behavior, shifting the plot further into the region fooddeficit>drinkdeficit (below the diagonal line). **Bottom row:** the asymmetric environment. Here there are often long periods searching for drink, so more time is spent in the region drinkdeficit>fooddeficit. *Bottom left*: the symmetric pleasure system. *Bottom center*: adding pleasure from drinking increases persistence in drinking and shifts the plot towards the region fooddeficit>drinkdeficit, partially correcting the imbalance between the deficits introduced by the asymmetry from the environment. *Bottom right*: adding pleasure to eating shifts the plot in the other direction, resulting in the drink deficit coming dangerously close to the fatal limit.

**Figure 18.**
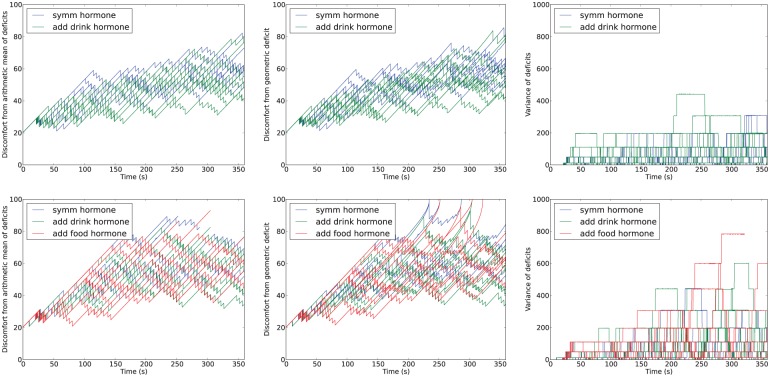
Time-series of metrics from Experiment 3. *Top*: in the symmetric environment (Conditions 1 and 2, equal availability of each resource); *bottom*: in the asymmetric environment (Conditions 3, 4 and 5, scarce drink resource).

**Figure 19.**
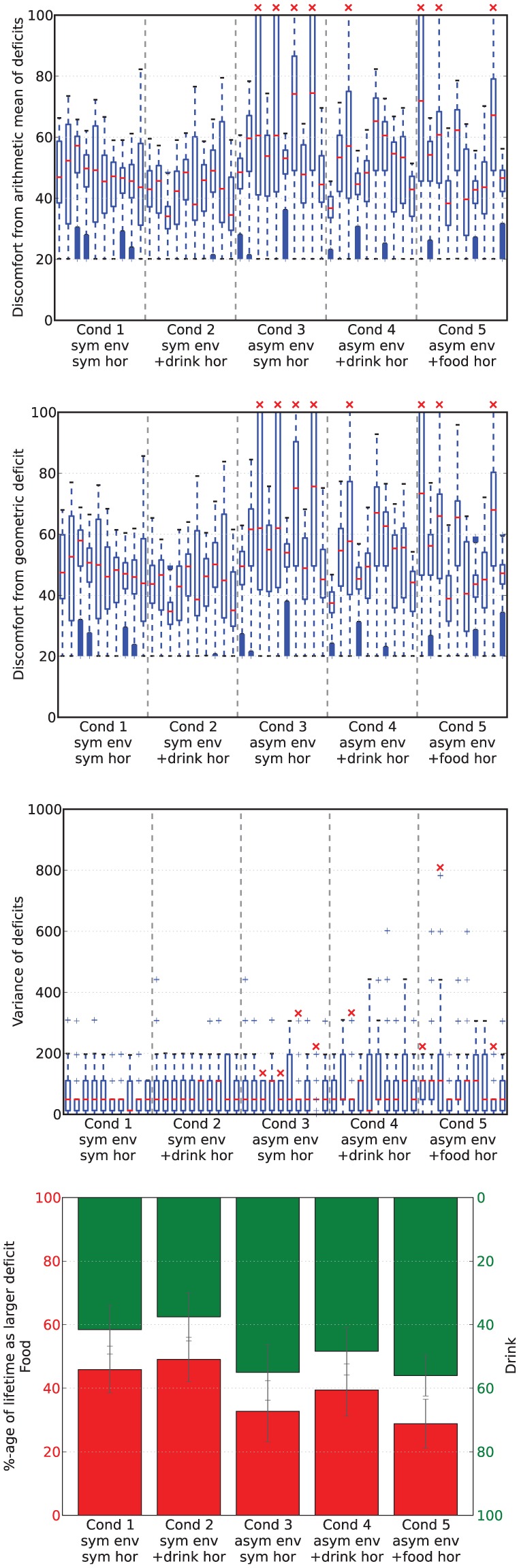
Metrics from runs in Experiment 3. In the box-and-whisker plots, the red lines show the medians while the boxes extend to the upper and lower quartiles of the data. The red crosses indicate those runs in which the robot died. The final plot gives an idea of the balance, in terms of time, between homeostatic deficits in each scenario, showing the percentage of the lifetime in which each of the deficits was larger. The lower bars (in red, read from bottom to top) show the percentage of time that the food deficit was larger, and the upper bars (in green, read from top to bottom) show the percentage of time that the drink deficit was larger.

**Figure 20.**
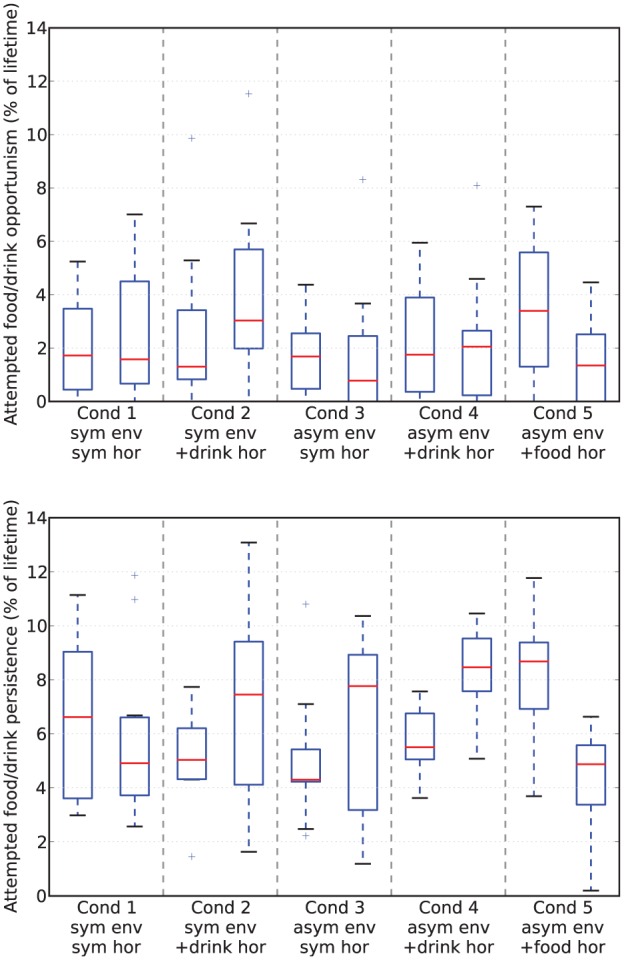
Rates of attempted opportunism (*top*) and persistence (*bottom*) from Experiment 3. Due to the asymmetric resource distribution of the environment we have separated opportunism/persistence directed at food (on the left in each pair) and at drink (on the right in each pair). Note that these are not directly comparable to the equivalent values for the previous experiments (the Moα condition in Figures 9 and 14) since we have decreased the nutritional value of each resource in order to increase the time taken for the robot to reach its satiation boundary.

Figure 17 shows deficit–space plots from example runs under each condition. Figure 18 shows the discomfort metrics for each run plotted against time and Figure 19 the distribution of the performance metrics for each run. Figure 20 shows rates of opportunism and persistence. Table 2 shows means of the geometric discomfort and of the variance of the deficits over all the runs. See captions for more detailed explanations.

**Table 2.** Means of, respectively, the arithmetic discomfort, the geometric discomfort and the variance of the deficits over all runs in each condition in Experiment 3.

	Symmetric env.			Asymmetric env.		
**Symmetric pleasure**	46.7	47.4	61.8	56.6	57.6	76.2
**Additional pleasure from drink**	42.5	43.4	81.5	48.8	50.1	98.0
**Additional pleasure from food**	—			50.9	52.2	105.5

Looking at Figures 19 and 18, we can see that there were no deaths in either of the two conditions for release of the hormone in the symmetric environment. In comparison, the asymmetric environment was clearly more challenging, with four deaths (two from thirst, two from hunger) occurring in the condition in which the hormone release depends only on the homeostatic variables, one death (from hunger) when there was additional hormone release upon drinking (additional hormone release linked to the scarce resource), and three deaths (two from thirst, one from hunger) with additional hormone release upon eating (additional hormone release linked to the abundant resource).

Looking at the rates of attempted persistence and opportunism (Figure 20) we can see that, as in the Moα condition in Experiments 1 and 2, the level of persistence is higher than the level of opportunism. Comparing the symmetric and asymmetric environments without the extra source of pleasure (Conditions 1 and 3, shown by the first and third pairs of Figure 20), there appears to be a small decrease in opportunism for the scarce resource (likely because these are less frequently encountered) and an increase in persistence for the same resource, although with an increased variance. This increased persistence linked to the introduction of asymmetry in the environment may be due to the robot having a higher deficit when it finds the resource, as this would make the perceptual component in equation (1) larger. Introducing the additional asymmetric pleasure from consuming a resource (Conditions 2, 4 and 5, shown by the second, fourth and fifth pairs in Figure 20) appears to increase the level of persistence for that resource, as expected. There also seems to be a small effect of this extra pleasure on opportunism, which increases for the pleasurable resource. This small effect may be due to the fact that the larger releases of hormone take longer to decay, leading to the presence of residual hormone in the system for a longer period after consumption.

Looking at Table 2, we can see that the additional pleasure hormone decreases discomfort (i.e. improves homeostatic management) but increases the variance of the deficits. These changes occur when the extra pleasure comes from eating either of the resources; although we expected this to happen when extra pleasure was associated with eating the scarce resource (drink), we had not anticipated that this would also happen when extra pleasure was associated with eating the plentiful food resource (Experimental Condition 5). Although the increase of variance introduced by the extra pleasure might in principle seem to indicate worsening of homeostatic management, this is not necessarily the case. To have a better understanding of how homeostatic balance is managed, we have also calculated the percentage of time during which each deficit is larger. Results are shown in the bottom plot of Figure 19. For each set of runs, the lower red bar shows the percentage of the lifetime of the robot during which food was the larger deficit, while the upper green bar shows the percentage of time during which the drink deficit was larger. In the symmetric environment with no additional pleasure (Experimental Condition 1, shown by the leftmost pair of bars), the two deficits are well balanced with respect to each other by this metric. The addition of extra pleasure for the drink resource (Experimental Condition 2, shown by the second pair of bars) results in a small shift of this balance so that the drink deficit is more often the smaller of the two deficits. The asymmetric environment, where the drink resource is scarce (Experimental Condition 3, shown by the third pair of bars), clearly shows an asymmetry in the balance of the deficits, with the deficit corresponding to the scarce resource (drink) more often being the larger of the two deficits. Additional pleasure from consuming one resource (Experimental Conditions 4 and 5, shown respectively by the fourth and fifth pair of bars) decreases the amount of time that the deficit corresponding to the more pleasurable resource is larger, as in the symmetric environment. When the extra pleasure is linked to the scarce drink resource (Condition 4, fourth pair) it has the effect of partially reducing the asymmetry caused by the environment; however, when the plentiful resource (food) is more pleasurable, it adds to the asymmetry caused by the environment.

## 7 Discussion

In the static and unchanging environment with readily available resources used in *Experiment 1*, if we only consider the viability metrics such as the discomfort (Figure 8), there is little to distinguish the robot with Modulated α from one with a fixed value of α, in particular the medium value. In fact, as we already observed in Section 4.3, the best behavior in terms of viability is to use a high value of α.

However, if we consider its rates of persistence and opportunism (Figure 9), our results show that the robot with modulated α behaves in a manner that could not correspond to any fixed value of α, since for fixed α the levels of persistence and opportunism are tightly linked, and increasing one will necessarily increase the other, whereas with Modulated α they show some independence. In the discussion of Experiment 2, we will see that this different manner of behaving can also result in improved viability in a more challenging environment.

Let us consider how the hormone-related mechanism contributes to this different behavior. No component in our system is explicitly controlling the rates of persistence and opportunism. However, the interaction between the following two elements gives rise to, on average, prolonged interaction with consumed resources.

- The hormone acts to modulate the perceptual salience of environmental cues, and therefore their incentive salience related to motivation.
- The interaction context in which the hormone is released—successful consumption of a resource to reduce a homeostatic deficit—means that the relevant resource is necessarily present, likely in a situation where it can be readily perceived and consumed.

Our mechanism thus increases attempted persistence but does not increase the likelihood of attempted opportunism. Attempted opportunism more often occurs in contexts where no resource has been consumed recently, and hence the hormone has fallen to its background level; this background level is what has a dominant influence on the level of opportunism. This is seen in the results of Experiments 1 and 2, in which the levels of opportunism for LFα and Moα were comparable (Figures 9 and 14). Recall that the low fixed α of 0.1 was equal to the α resulting from the background level of hormone in the Moα condition (Section 4.2).

In the more challenging environment with difficult access to resources used in *Experiment 2*, we observe more interesting differences across the α conditions tested in terms of viability metrics. In particular, the fact that the condition with the highest average comfort (HFα) is not the same as the condition with the lowest mortality (MFα) deserves some comment. Since death depends only on the larger of the two homeostatic variables, it is linked not only to the average of the deficits but also to their variance. For high α values, the large variance is linked to the higher levels of persistence and opportunism—both phenomena mean that the robot is acting to reduce the smaller of its two deficits, thus increasing the variance. This can be viewed as a certain type of ‘risk-taking’, since it allows the larger of the deficits to edge closer to the critical boundary. In this environment, in which resources may take some time to locate, this leads to the increased risk of death that we see with large α values.

Note that the Moα condition has not been optimized in any way. That is, the values associated with the level of the hormone (rate of release, background level, decay rate), and the scaling factor between hormone level and α were not tweaked to give better performance (see Sections 2.5 and 4.2 for information about how the values were chosen). Hence, the fact that Moα does not perform best in this experiment does not mean that a modulated α will always be out-performed by some value of fixed α. In fact, we argue below that using a modulated α has more potential than a fixed α.

Characterizing the robot’s behavior in this environment in terms of persistence and opportunism in the Moα condition compared to constant α conditions builds on the results obtained in Experiment 1. In Figure 14 we can see that, as in Experiment 1, for the three fixed α conditions, the rates of both attempted persistence and opportunism are related to each other as both increase with increasing values of α. In contrast, for the Moα condition, the rate of opportunism is comparable to the rate for LFα and the rate of persistence is comparable to the rate for MFα. However, unlike in Experiment 1, in this more complex environment, increasing the value of a fixed α does not necessarily give the best result in terms of viability. In particular, too much opportunism is maladaptive as it increases the time taken for the robot to find the most needed resource (as seen in the runs with HFα, Figure 13). Too low a level of persistence is also maladaptive, since then the robot moves on too early from a resource that it is consuming, without taking sufficient advantage of it, and this leads to both deficits increasing over time, and eventual death (as seen in the runs with LFα).

As we have already mentioned, with fixed values of α the rates of persistence and opportunism are strongly linked. However, with α modulated by our pleasure mechanism, because α varies with the context of the interaction (consuming or exploring) in a way that discriminates between persistence and opportunism, the rates at which these two phenomena occur are more independent. Specifically, the background level of the hormone relates to the level of opportunism, while the level of the hormone during consumption (as a result of the interaction between the rate of hormone release and the decay rate of the hormone) determines the level of persistence. This separation, allowed by our pleasure system, has important consequences from the point of view of the design of the robot. In particular, in provides the ‘designer’ (e.g. a human or an evolutionary algorithm) and the robot with a mechanism to increase the level of persistence while maintaining opportunism at a lower level (determined by the background level of the hormone) as required by different environments or changing circumstances. In other words, it introduces a useful mechanism for adaptation to the environment.

**Figure 21.**
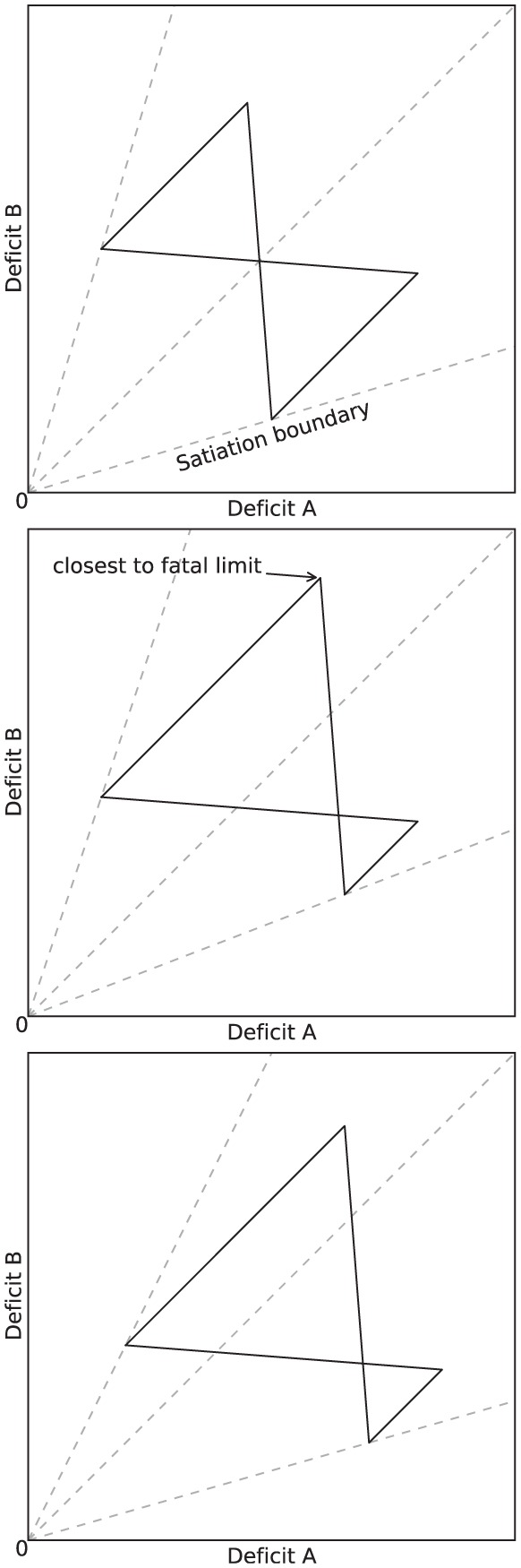
Idealized activity cycles in the deficit space. *Top*: equal availability of both resources and equal persistence leads to symmetric cycles. *Center*: Resource B is less available and takes longer to locate, the long search period leading to the agent coming closest to death. *Bottom*: A larger persistence on Resource B than Resource A shifts the cycle away from the fatal limit; equal amounts of each resource are still consumed, but the agent continues to Resource B further beyond the point where DeficitA=DeficitB (i.e. greater persistence). Note that the length of the line does not correspond to time as the consumption of resources causes rapid changes in the deficits compared to their slow growth during searching.

The effects that introducing asymmetry in the environment and in the release of pleasure have on the behavior and viability of the robot (*Experiment 3*) are clearly visible in our metrics. To describe these effects, let us consider the effects that these types of asymmetry have on an idealized activity cycle, depicted in Figure 21.^8^ The activity cycle at the *top* shows the behavior cycle in a static and symmetric environment, as reflected by the dynamics of the internal deficits. The activity cycle in the *center* shows the effect of introducing asymmetry in the number of resources of each type: this results in more time spent searching for the scarce resource (corresponding to Deficit B in the figure). Survival is more problematic in this case, as Deficit B comes closer to the fatal limit. The activity cycle at the *bottom* shows the effect of introducing asymmetric pleasure on top of the asymmetry of the environment. While more time is spent searching for the scarce resource, the whole cycle is shifted away from the fatal limit, showing that this pleasure improves the viability, and hence the survival, of the robot. Note that, to an observer of the behavior of the robot with no access to its internal state, the behavior giving rise to the activity cycles on the *center* and the *right* of Figure 21 would appear identical—the robot spends exactly as much time searching and consumes equal amounts of each resource. However, the difference for the robot’s wellbeing is significant as the cycles occur in different places in the deficit space. This difference is also reflected in our persistence metric, the computation of which requires knowledge of the internal deficits.

As we noted in Section 6.3, Table 2 shows that, in each environment, the additional source of pleasure results in: (a) a decrease in (arithmetic and geometric) discomfort (i.e. an increase in comfort) and (b) increased variance between the deficits. These two effects occur even if the extra pleasure happens when consuming the plentiful resource, which intuitively is the ‘wrong’ resource in terms of improving adaptation to the environment. Both effects can be explained by the fact that the additional pleasure increases consumption of the more pleasurable resource, which leads to a large difference between the two deficits; hence, when the less pleasurable resource is eventually consumed, more of it is consumed, as its associated deficit remains the pressing deficit for longer. Due to this, the robot is more frequently in a state where the difference between its deficits is large (increasing variance) and consumes more of both resources (decreasing discomfort by reducing both deficits).

Although increased variance might seem to indicate a worse ‘homeostatic balance’, this is not necessarily the case, since variance is not the only way to conceptualize balance in the system. In order to gain a deeper understanding of the behavior of our robot, we calculated how long each deficit was the larger as a proportion of the lifetime of the robot (Figure 19). We can see that the symmetric environment and symmetric pleasure (Condition 1) leads to roughly equal temporal balance between the two deficits, as would be expected. Adding extra pleasure linked to consumption of one of the symmetric resources (drink, Condition 2) slightly reduces the time for which the deficit associated with that resource is larger. In the asymmetric environment, the temporal balance is shifted by a large amount, and therefore the deficit associated with the scarce resource (drink) is the larger deficit for most of the time. The addition of a second source of pleasure from consuming the scarce resource helps to mostly restore this balance, while added pleasure from consuming the abundant resource slightly increases the imbalance. These shifts in the balance between the two deficits can also be seen in the shifts in the example deficit space plots (Figure 17). Thus the extra ‘asymmetric’ pleasure provides another useful mechanism for adaptation to the environment. In the discussion of Experiment 2, we saw how the pleasure system related to the satisfaction of needs provides a mechanism to control persistence and opportunism that can be used by either the designer or the robot itself to adapt to different environmental circumstances. In the same vein, the additional pleasure introduced in Experiment 3 provides a mechanism to counteract an asymmetry in the temporal balance between the homeostatic variables caused by different environments or changing circumstances.

Let us finally discuss our choice of mechanism to introduce asymmetry in the release of the pleasure hormone. In this experiment, additional sensory pleasure, unrelated to the nutritional value of the resources, was released on successful execution of the consummatory behavior. However, it is worth noting that, in our environment, a similar amount of additional pleasure could have been achieved by increasing *s* (the scaling factor connecting the second derivative of the deficits with the release of hormone in equation (2)) for just one of the deficits. Since our resources all have the same nutritional value, it would be possible to choose a value for *s* such that the amount of hormone released was the same as the additional pleasure. It is then worth asking what the difference between these two mechanisms—adding a second source of pleasure or changing the value of the *s* parameter—is.

In the first place, in an environment where resources could reduce deficits by different amounts, the two systems would differ in that a simple change of *s* would also scale the difference in the nutrition; therefore, if one resource gave half the nutrition of another, then it would result in half the release of hormone per bite, reducing the persistence in consuming this resource. This is potentially maladaptive in some environments, as we have seen in Experiments 1 and 2. With the additional pleasure mechanism, only one of the sources of pleasure—the pleasure due to the nutritional value—would be halved. The other source of pleasure would change in a way determined by the mechanism used to trigger the release of the pleasure hormone; for example, if the trigger were simply the act of eating, then there would be no change. This separation of the two sources of pleasure presents advantages, such as the ability to discriminate between highly nutritious and less nutritious resources, or the better maintenance of persistence for less nutritious resources, which might be vital in some environments. However, this separation also comes at a cost, as it means that the robot could find a resource that is very pleasurable to eat but has little benefit in terms of the homeostatic variables.

Second, the two mechanisms show a difference in the complexity of the phenomenon that they can model. Changing the scaling factor *s* corresponds to a very simple pleasure mechanism only linked to nutrition. Compared to this straightforward model, the additional pleasure mechanism offers the potential for more complex roles for pleasure that appear in evolutionarily more complex organisms. It could, for example, allow for a system in which a resource that has not been consumed for some time would give more pleasure than if it had been consumed recently, or for integrating cultural differences in taste-related pleasure.

Third, there is a difference in the temporal dynamics of the two pleasure mechanisms. In our model, consumption of a resource quickly results in a drop in the corresponding homeostatic deficit, and hence nutritional pleasure is an immediate ‘reward’ that can be clearly associated with the ‘eating’ behavior that led to the correction of the deficit. However, in organisms with more complex digestive systems that take time to process food, there is a delay between the eating behavior and the drop in deficit, and hence the nutritional pleasure is delayed (we could talk of a ‘delayed reward’ in terms of reinforcement learning) since it only happens upon digestion of the resource. Changing the scaling factor cannot model these more complex systems, whereas having two sources of pleasure hormone release related to consumption opens the door to keeping an immediate pleasurable reward related to consumption alongside a more delayed pleasurable signal associated with nutrition.

## 8 Conclusion and future work

We have presented a basic model of pleasure and investigated its effect on the decision making of a motivated autonomous robot. Unlike other work that had looked at pleasure in the context of relatively high-level functions such as learning and memory, we think that pleasure can already play an important role at a more basic level. Therefore, we have focused on lower cognitive functions and investigated the interaction between pleasure, perception and motivation, in particular the incentive salience of survival-related external stimuli. By affecting incentive salience, our pleasure fosters continuing the ongoing interaction, which is one of the main functions attributed to pleasure in the literature. However, we have not taken for granted the other main function often attributed to pleasure: signaling the usefulness of stimuli. We have thus considered two types of pleasure, a well-being related pleasure directly linked to the satisfaction of survival-related needs, and a purely ‘sensory’ pleasure (hedonic quality) unrelated to the satisfaction of needs.

We have framed our study in the context of a classical two-resource AS problem to investigate the effect of these types of pleasure on the viability and decision-making behavior of the robot. We have conducted three sets of experiments varying the following aspects to create increasingly complex AS problems: the availability of (easy or difficult access to) resources and their (symmetric or asymmetric) distribution in the environment, and how the release of pleasure relates to their consumption—either to the ‘nutritional value’ or simply to the act of consuming.

Our results indicate that pleasure, including pleasure unrelated to need satisfaction, has value for homeostatic management in terms of improved viability, as well as in terms of more flexibility in adaptive behavior. Regarding the latter, this is the case specifically in situations where opportunism has a penalty, but increased persistence is beneficial, and where an asymmetry in the availability of resources results in the need to consume each of the resources in different ways in order to achieve good management of homeostasis. Regarding viability, the extent to which the different ‘types’ of pleasure are adaptive or maladaptive depends on the features of the environment and the demands it poses on the task, in addition to the ‘metabolism’ of the robot. In Experiment 1, simply maximizing pleasure (regardless of whether it is related to need satisfaction or not) improved viability. In Experiment 2, constant moderate pleasure (unrelated to need satisfaction) gave the best viability; the pleasure released as a function of need satisfaction was comparable to this by some of the metrics, but additionally more flexible in terms of the behavior of the robot, notably the possibilities it offers to manage persistence and opportunism independently, and hence to display them in the appropriate context. In Experiment 3, in environments with asymmetric availability of resources, the addition of ‘purely sensory’ (not related to need satisfaction) pleasure associated with the scarce resource improved viability.

The work presented here is a first step towards an incremental study of the role of pleasure in AS. The next steps will include the inclusion of resources with different nutritional values, the introduction of dynamic elements in the environment, as well as integrating this model in our social robot Robin.

## Notes

1. This framework was initially developed to implement the more complex architecture of Robin. We have used it in the present architecture, which includes a subset of Robin’s, but also adds the more complex pleasure model presented here that we aim to eventually integrate in Robin.

2. In AS architectures without the self-imposed constraint of keeping the number of elements to the strict minimum of two for the purposes of analysis, each motivation could be satisfied by a number of behaviors providing different approaches or strategies to solve the problem of satisfying needs.

3. We use the negative second derivative, rather than the second derivative, because we are using the deficit rather than the level of the homeostatic variables.

4. ‘Extended behaviors’ are ongoing behaviors that execute in their own thread and may last longer than a single tick of the behavior selection loop.

5. This information is added to the list of actuators that are in use by extended behaviors, which might go beyond this specific behavior selection cycle.

6. The ‘arithmetic mean’ is the most widely used mean, familiar to most people from high-school. It is normally referred to simply as the ‘mean’; however, we wish to avoid ambiguity, as we also use the geometric mean.

7. Further informal tests in this environment appeared to show that, even with much higher values of α, the robot would continue to manage its homeostatic variables well.

8. Note that these activity cycles are highly idealized. In our experiments, there is great variation in the amount of time that it takes the robot to find a resource, particularly the scarce resource. This makes the availability of the resources unpredictable, and therefore real activity cycles are much less regular.
